# Role of dietary factors in the prevention and treatment for depression: an umbrella review of meta-analyses of prospective studies

**DOI:** 10.1038/s41398-021-01590-6

**Published:** 2021-09-16

**Authors:** Yujie Xu, Linan Zeng, Kun Zou, Shufang Shan, Xiaoyu Wang, Jingyuan Xiong, Li Zhao, Lingli Zhang, Guo Cheng

**Affiliations:** 1 Department of Nutrition, Food Safety, and Toxicology, West China School of Public Health and West China Fourth Hospital, Sichuan University, Chengdu, China; 2 Department of Pharmacy, Evidence-Based Pharmacy Center, Key Laboratory of Birth Defects and Related Diseases of Women and Children (Sichuan University), West China Second University Hospital, Sichuan University, Chengdu, China; 3 Department of Health Policy and Management, West China School of Public Health and West China Fourth Hospital, Sichuan University, Chengdu, China; 4 Laboratory of Molecular Translational Medicine, Center for Translational Medicine, Key Laboratory of Birth Defects and Related Diseases of Women and Children (Sichuan University), Ministry of Education, Department of Pediatrics, West China Second University Hospital, Sichuan University, Chengdu, China; 5 Healthy Food Evaluation Research Center, West China School of Public Health and West China Fourth Hospital, Sichuan University, Chengdu, China

**Keywords:** Scientific community, Depression

## Abstract

The role of diet in depression is becoming increasingly acknowledged. This umbrella review aimed to summarize comprehensively the current evidence reporting the effects of dietary factors on the prevention and treatment of depression. PubMed, Embase, and the Cochrane Library were searched up to June 2021 to identify relevant meta-analyses of prospective studies. Twenty-eight meta-analyses, with 40 summary estimates on dietary patterns (*n* = 8), food and beverages (*n* = 19), and nutrients (*n* = 13) were eligible. The methodological quality of most meta-analyses was low (50.0%) or very low (25.0%). Quality of evidence was moderate for inverse associations for depression incidence with healthy diet [risk ratio (RR): 0.74, 95% confidential interval (CI), 0.48–0.99, *I*^2^ = 89.8%], fish (RR: 0.88, 95% CI, 0.79–0.97, *I*^2 ^= 0.0%), coffee (RR: 0.89, 95% CI, 0.84–0.94, *I*^2 ^= 32.9%), dietary zinc (RR: 0.66, 95% CI 0.50–0.82, *I*^2 ^= 13.9%), light to moderate alcohol (<40 g/day, RR: 0.77, 95% CI, 0.74–0.83, *I*^2 ^= 20.5%), as well as for positive association with sugar-sweetened beverages (RR: 1.05, 95% CI, 1.01–1.09, *I*^2 ^= 0.0%). For depression treatment, moderate-quality evidence was identified for the effects of probiotic [standardized mean difference (SMD): −0.31, 95% CI, −0.56 to −0.07, *I*^2 ^= 48.2%], omega-3 polyunsaturated fatty acid (SMD: −0.28, 95% CI, −0.47 to −0.09, *I*^2^ = 75.0%) and acetyl-l-carnitine (SMD: −1.10, 95% CI, −1.65 to −0.56, *I*^2^ = 86.0%) supplementations. Overall, the associations between dietary factors and depression had been extensively evaluated, but none of them were rated as high quality of evidence, suggesting further studies are likely to change the summary estimates. Thus, more well-designed research investigating more detailed dietary factors in association with depression is warranted.

## Introduction

Depression, characterized by sadness, hopelessness, lack of interest, low self-worth, and recurrent thoughts of death, is highly prevalent among the general population and affects over 320 million individuals worldwide [1, 2]. It can heavily weaken sufferer’s ability to cope with work and destroy their daily life skills, impose a rising burden on their families and caregivers, as well as increase health care service costs [3]. At its worst, depression can eventually lead to disability or premature death [4]. As the World Health Organization reported [5], depression was the primary reason for disability and a major cause for the global burden of disease. Depression has thus become an important public health concern and investigation on the prevention and management of this disease has turned into a priority.

The pathophysiology of depression is still vague, but existing evidence suggests that it is a complicated disease caused by the interaction of genetic, biological, and environmental factors, likely involving several mechanisms [6, 7]. Although genetic and biological factors as unmodifiable factors partly play a role in the pathology of depression, modifiable factors such as environmental factors (including lifestyle, diet, and social support) contribute to the onset of the disease as well [8–11]. There has been a growing body of research exploring the associations between dietary factors and depression [12, 13]. In the last decades, many systematic reviews and meta-analyses have concluded evidence on the associations between dietary patterns or dietary quality indices, food groups, macronutrients, and micronutrients, and the incidence of depression or the severity of depressive symptoms. These findings could be of importance for the prevention and treatment of depression. However, the strength, precision, and quality of the evidence, and potential bias of the associations in these systematic reviews and meta-analyses still need to be clarified.

Umbrella review is a useful literature tool to provide a broad overview of published evidence in systematic reviews and meta-analyses on a certain topic. They can reveal the strength of the estimates and the certainty of the conclusions, as well as evaluate the influence of potential bias of the associations. Recent reports summarized evidence for selected dietary factors on the prevention of depression [14–17]. Strong evidence was found for a decreased incidence of depression with higher consumption of tea and dietary zinc [14], and high adherence to Mediterranean diet and healthy diet [15, 17], as well as an increased incidence of the disease with high adherence to a pro-inflammatory diet [15]. Sanhueza et al. summarized convincing evidence and found a decreased incidence of depression with a higher intake of olive oil, fish, folate, and omega-3 fatty acids [16]. However, none of these studies focused on any existing evidence between dietary factors and the incidence of depression among the general adult population, and few of them summarized dietary factor interventions for depression treatment. Meanwhile, it remains to assess the methodological quality of the meta-analyses and quality of evidence by validated tools. Therefore, we aimed to review comprehensively and appraise the current best evidence regarding the preventive or therapeutic effects of dietary factors (i.e., dietary patterns, food groups, food and beverages, macronutrients, and micronutrients) on depression among healthy or depressed adults.

## Methods

The current umbrella review was conducted and reported in line with the Preferred Reporting Items for Systematic Reviews and Meta-Analyses (PRISMA) checklist [18]. Our protocol was prospectively registered at the PROSPERO—the International Prospective Register of Systematic Reviews (ID: CRD42021227811).

### Search strategy and study selection

PubMed, Embase, and the Cochrane Library were searched from database inception until July 20, 2021, to identify meta-analyses investigating the associations between dietary exposures and depression incidence and the effects of dietary interventions on the severity of depressive symptoms. Medical subject heading (MESH) terms and keywords used for each database included “diet”, “dietary patterns”, “food”, “food group”, “food and beverage”, “nutrient”, “depression”, and “meta-analysis”. Additional detailed search strategies were presented in Supplementary Table 1. No restrictions or filters were applied. We also screened references cited in the relevant meta-analyses by manual. The titles, abstracts, and full texts were evaluated by two authors (YJX and LNZ), and any discrepancies were decided by consensus.

We included studies if the following inclusion criteria were met: systematic reviews with meta-analyses included ≥2 prospective cohort studies or randomized controlled trials (RCTs) that investigated the associations between dietary factors (i.e., dietary patterns, foods and beverages, nutrients, and phytochemicals) and depression incidence or the severity of depressive symptoms; conducted in general population aged 18 years or older who behaved well mental health, were at risk of depression (i.e., high levels of psychological mood biomarkers, sub-clinical symptomatology symptoms or vulnerability to mood disorders) or were diagnosed with depression; the pooled estimate sizes [i.e., odds ratio (OR), risk ratio (RR), hazard ratio (HR), mean difference (MD), or standardized mean difference (SMD)] with their 95% confidence interval (CI) were calculated.

Studies were excluded if they were only conducted among children, teenagers, and lactating/pregnant women; used a network meta-analysis method; considered bipolar disorder, secondary depression as the outcome; concentrated on plasma nutrient levels or biomarkers rather than dietary intakes. A meta-analysis that pooled analysis of cohorts with individual data was not eligible for this umbrella review. If a meta-analysis included both prospective and retrospective studies, only primary studies of incident cases had been included. When more than one meta-analysis was presented in an article, we included all meta-analyses and assessed them separately following inclusion criteria. For multiple meta-analyses reporting on the same dietary factor and the outcome, we included the one with the largest number of primary studies. While the meta-analysis with the largest number of participants was chosen when multiple meta-analyses included the same number of studies.

### Data extraction

Data were extracted initially by one author (YJX) using a pre-designed form and validated by another author (KZ). For each meta-analysis, the following information was extracted: (1) characteristics of included publications, including first author, journal, publication year, country of the corresponding author, database searched and search period, number and type of included primary studies, as well as quality assessment score; (2) characteristics of the study population, including sample size, mean age and sex, type of interested exposure, intervention, comparison, and outcome; (3) results of meta-analyses, including meta-analysis method, dose-response analysis, pooled estimate size with their 95% CI, heterogeneity and publication bias. For meta-analyses that comprised not only general adults but also children, teenagers, or pregnant women, we only included the effect size calculated based on general adults.

Two researchers (XYW and SFS) independently extracted the following data from primary studies included in each meta-analysis: the first author’s name, year of publication, exposure (including the dose of exposure), number of participants and cases, sex and age of participants, and effect size that adjusted for most confounders, along with their 95% CI, as well as the adjustment factors included in the model. Any discrepancies were settled by consensus.

### Methodological quality assessment and evaluation of the quality of evidence

A Measurement Tool to Assess Systematic Reviews version 2 (AMSTAR-2) checklist was applied to assess the methodological quality of the included meta-analyses [19], which contained 7 critical domains and 9 non-critical domains to capture review quality and confidence. For each item, the answer could be “yes”, “no”, or “partly yes”. Overall quality was rated as (1) high, no or one non-critical weakness; (2) moderate, more than 1 non-critical weakness; (3) low, 1 critical flaw with or without non-critical weakness; (4) and very low, more than 1 critical flaw with or without non-critical weakness.

The quality of evidence of included meta-analyses was evaluated using the NutriGrade [20]. It was a numerical scoring system with scores ranging from 0 to 10 points, which comprised 8 items, i.e., risk of bias and study quality of the primary study, estimate precision, heterogeneity, directness, publication bias, funding bias, effect size, and dose-response association. The level of evidence was judged by four categories: (1) high quality, a score ≥8 points, so a reliable effect estimate may not change in further research; (2) moderate quality, a score of 6 to <8, indicating an effect estimate with a moderate confidence and would be changed by further investigation; (3) low quality, a score of 4 to <6, where a likelihood that further studies would change the effect estimate; (4) very low quality, a score less than 4 indicated that there was very limited and uncertain evidence. All the assessments were conducted independently by 2 authors (YJX and LNZ) and any discrepancies were resolved by consultation to a senior reviewer (GC).

### Statistical analysis

We reanalyzed all the pooled estimates and their corresponding 95% CI in included meta-analyses using a random effect model, to ensure only prospective studies were pooled and all relevant measurements, e.g., heterogeneity evaluation, were consistent. As for primary studies that reported multiple estimate sizes separately for different age, race, or sex, a fixed effect model was applied to generate summarized effect size before the overall meta-analysis. We recalculated dose-response meta-analysis if the estimate for each primary study was reported separately, otherwise, we extracted the adjusted summary effect size from the published meta-analyses. Pooled effect size of each dietary factor was presented in a forest plot. Moreover, we calculated *I*^2^ as a measure of heterogeneity between studies. We assessed publication bias of meta-analysis with ≥5 primary studies by using the funnel plot and Egger’s test. All statistical analyses were performed with Stata Version 14.0.

## Results

**Fig. 1:**
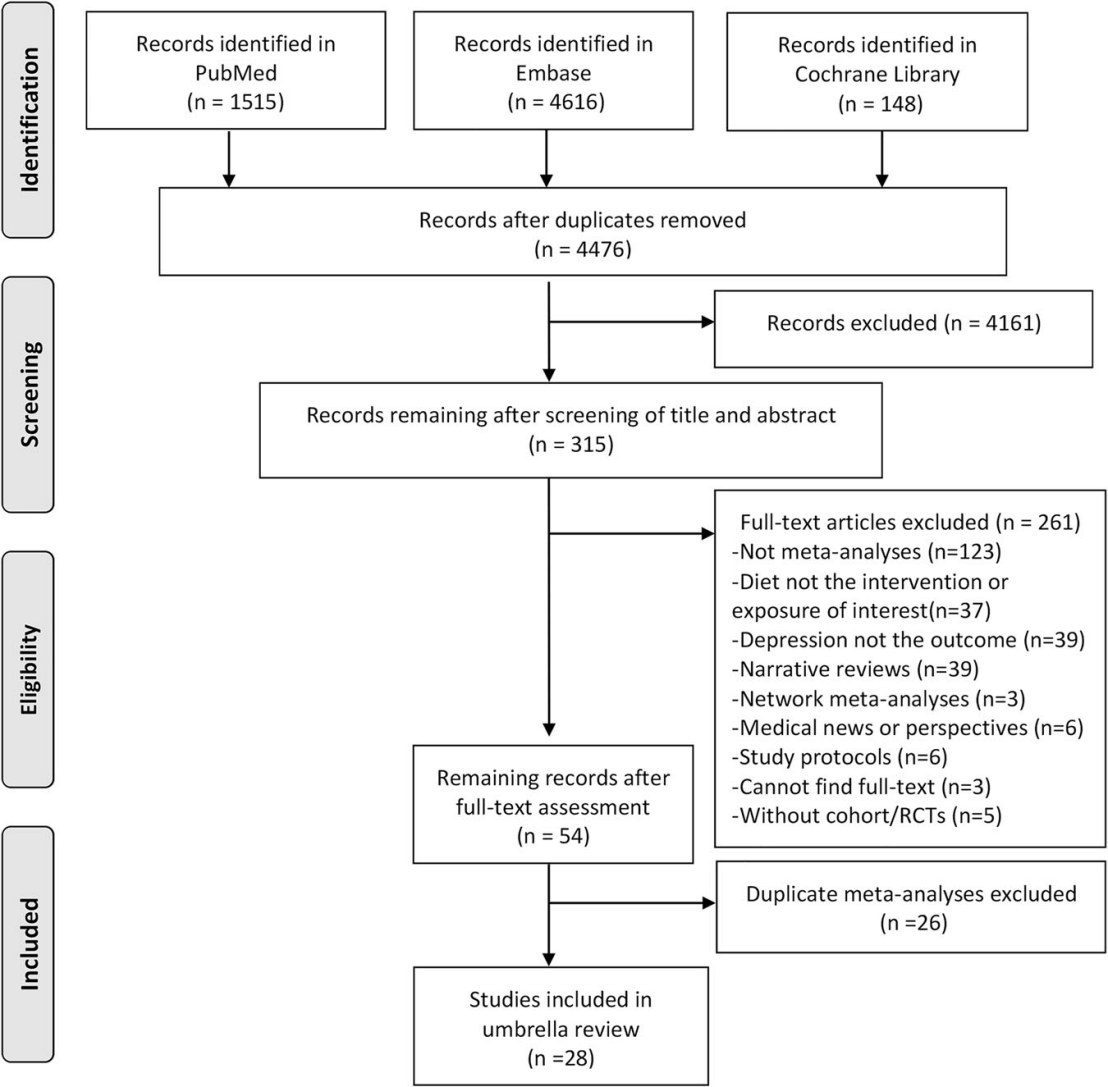
Flow chart illustrating the literature search process in the umbrella review.

Our literature search identified 4476 publications after 1803 duplicates were removed. The flow chart summarizing study selection and reasons for exclusion was presented in Fig. 1. After screening for 315 full-text articles, 261 articles were excluded according to our exclusion criteria. We identified more than 1 meta-analyses for the same exposures or interventions, and we included the recent meta-analyses with the largest number of primary studies or participants. Finally, 28 meta-analyses with 40 reanalyzed effect sizes on dietary patterns or dietary quality indices (*n* = 8), food groups (*n* = 19), nutrients (*n* = 13), regarding preventive or therapeutic effects on depression, were eligible for this umbrella review.

We found meta-analyses focusing on the following exposures or interventions: alternate healthy eating index (AHEI) or AHEI-2000 [21], vegetarian diet [22], dietary inflammatory index (DII) [23], Mediterranean diet [24], healthy dietary pattern [25, 26], western diet [27], very low-calorie diet [28], and ultra-processed foods [29], cocoa-rich foods [30], red or processed meat [31], alcohol drink [32], sugar-sweetened beverages (SSBs) [33], fish [34], fruits [35], vegetables [35], coffee [36], tea [37], caffeine [38], prebiotics [39], probiotics [39], and dietary zinc [40], dietary magnesium [41], n-3 PUFA [42–44], acetyl-l-carnitine (ALC) [45], as well as vitamin D [46], folic acid [47], and total B vitamins [48].

### Characteristics of included meta-analyses

The characteristics of included meta-analyses in this umbrella review were shown in Table 1 and Supplementary Table 2. The year of the included meta-analyses published were between 2016 and 2021. The number of datasets searched ranged from 2 to 8. Most corresponding authors were from China (21.4%), followed by United Kingdom (17.9%), Italy (17.9%), Iran (14.3%), Australia (7.1%), South Korea (7.1%), Netherlands (3.6%), USA (3.6%), Spain (3.6%), and Canada (3.6%). While the primary studies included in these meta-analyses were conducted in Europe (51.3%), Asia (17.9%), North America (15.4%), Oceania (14.1%), and South America (1.3%). These meta-analyses pooled 2–26 primary studies, with the number of participants varied from 293 to 316,894. The percentage of enrolled male participants ranged from 0 to 100% with the mean age ranged from 18 to 95 years. Based on all meta-analyses included observational studies, the most important confounders in the associations between dietary factors and incidence of depression included age, sex, educational level, smoking, body mass index (BMI), physical activity, and other dietary factors, including total energy intake and alcohol consumption. According to the literature, the multiple logistic regression model and Cox proportional hazard regression model were used for adjusting confounders in 56% and 30% of the primary studies, respectively.

**Table 1:** Descriptive characteristics of the included meta-analyses in this umbrella review.

Author (year)	Dietary intervention/exposures	Comparison	Database searched	No. of studies	Type of included studies	Settings	Participants	Ages	%Male	Total *N*	Follow-up (years)	Country of author	AMSTAR-2
Preventive effect of dietary factor-dietary pattern													
Askari, 2020 [22]	Vegetarian diet	Highest vs lowest adherence	3	3	Cohort	2 EU;1 Asia	General adults	NR	0–100	19,783	4–7	Iran	Low
Lassale, 2019 [21]	AHEI or AHEI-2010 scores	Highest vs lowest adherence	3	3	Cohort	EU	General adults	37–61	NR	45,533 (3477 cases)	5–8.5	United Kingdom	Moderate
Tolkien, 2019 [23]	DII scores	Highest vs lowest adherence	2	5	Cohort	4 EU; 2 USA; 1 Australia	General adults	26–81	0–75	77,420	5–12.6	United Kingdom	Very low
Shafiei, 2019 [24]	Mediterranean diet	Highest vs lowest adherence	5	4	Cohort	2 EU;1 Australia;1 USA	General adults	49.5–69	NR	31,742	8–12.6	Iran	Low
Molendijk, 2018 [26]	Healthy dietary pattern	Highest vs lowest adherence	3	9	Cohort	3 EU; 2 Australia;1 USA; 2 Canada;1 Asia	General adults	18–74	0–75	105,494	6.5 months– 12.7	Netherlands	Moderate
Li, 2017 [27]	Western dietary pattern	Highest vs lowest adherence	2	8	Cohort	2 Asia; 3 EU;1 Australia; 1 Canada; 1 USA	General adults	20–77	NR	75,481	NR	China	Low
Preventive effect of dietary factor-food and beverages													
Pagliai, 2021 [29]	Ultra-processed foods	Highest vs lowest intake	5	2	Cohort	2 EU	General adults	18–86	NR	41,637 (2995 cases)	NR	Italy	High
Nucci, 2020 [31]	Red and processed meat	Highest vs lowest intake	3	5	Cohort	1 Asia;3 EU;1 Australia	General adults	39–81	NR	21,486 (2352 cases)	3–9	Italy	Low
Li, 2020 [32]	Alcohol rink	Heavy drinking (å 48 g/d) vs non-drinker	3	24	Cohort	12 EU; 6 USA; 5 Australia; 1 Canada	General adults	18–99	0–100	295,477 (21,378 cases)	1–40	China	Low
Li, 2020 [32]	Alcohol rink	Light-moderate drinking (<40 g/d) vs non-drinker	3	11	Cohort	NR	General adults	18–99	0–100	3423 cases	1–40	China	Low
Hu, 2019 [33]	Sugar-sweetened beverages	Highest vs lowest intake	2	4	Cohort	2 EU; 2 Australia	General adults	35–71	NR	277,405 (12630 cases)	NR	China	Low
Hu, 2019 [33]	Sugar-sweetened beverages	per 2 cups/d of cola	2	4	Cohort	2 EU; 2 Australia	General adults	35–71	NR	277,405 (12630 cases)	NR	China	Low
Yang, 2018 [34]	Fish	Highest vs lowest intake	2	8	Cohort	3 USA; 2 Australia; 2 Asia; 1 EU	General adults	25–82	0–100	101,443 (5732 cases	5–25	South Korea	Low
Yang, 2018 [34]	Fish	1 Serving/ week increment	2	3	Cohort	1 EU; 1 Australia; 1 Asia	General adults	45–82	0–100	35,431 (1136 cases	5–25	South Korea	Low
Saghafian 2018 [35],	Fruit	Highest vs lowest intake	5	6	Cohort	1 Asia; 2 EU; 2 Australia; 1 USA	General adults	21–85	NR	99,224 (3726 cases)	NR	Iran	Low
Saghafian, 2018 [35]	Fruit	Per 100 g increment	5	3	Cohort	1 Asia;1 Columbia;1 Australia	General adults	50–79	NR	78,855 (2593 cases	NR	Iran	Low
Saghafian, 2018 [35]	Vegetable	Highest vs lowest intake	5	7	Cohort	2 Asia;1 Columbia;2 Australia;2 UK	General adults	21–85	NR	100,295 (2928 cases)	NR	Iran	Low
Kang, 2018 [37]	Tea	Highest vs lowest intake	2	5	Cohort	3 Asia; 1 EU; 1 USA	General adults	42–93	0–100	259,818 (11,937 cases)	NR	South Korea	Very low
Grosso, 2016 [36]	Coffee	Highest vs lowest intake	2	3	Cohort	2 USA; 1 EU	General adults	NR	NR	316,894 (4656 cases	NR	Italy	Low
Grosso, 2016 [36]	Coffee	500 ml/d	2	3	Cohort	2 USA; 1 EU	General adults	NR	NR	316,894 (4656 cases	NR	Italy	Low
Wang, 2016 [38]	Caffeine	Highest vs lowest intake	4	4	Cohort	3 EU; 1 USA	General adults	>18	NR	29,033	NR	China	Very low
Wang, 2016 [38]	Caffeine	509 mg/day	4	2	Cohort	3 EU; 1 USA	General adults	>18	NR	5992 (669 cases)	NR	China	Very low
Preventive effect of dietary factor-nutrients													
Yosaee, 2020 [40]	Dietary zinc	Highest vs lowest intake	4	4	Cohort	3 Australia; 1 EU	General adults	å 54.7	NR	15,852 (2243 cases)	3–20	Iran	High
Deane, 2019 [42]	n-3 PUFA	Higher intake vs lower intake	5	13	RCTs	7 UK; 5 USA; 1 Asia	General adults	50–85	NR	26,528 (1355 cases)	NR	United Kingdom	High
Li, 2017 [41]	Dietary magnesium	Highest vs lowest intake	8	2	Cohort	EU	General adults	37–62	NR	15,259	6.3–20	China	Very low
Li, 2017 [41]	Dietary magnesium	361 mg/day	8	2	Cohort	EU	General adults	37–62	NR	15,259	6.3–20	China	Very low
Grosso, 2016 [43]	n-3 PUFA	Highest vs lowest intake	4	4	Cohort	3 EU;1 USA	General adults	24–69	NR	41,588	2–13	Italy	Low
Grosso, 2016 [43]	n-3 PUFA	1.8 g/d	4	2	Cohort	2 Australia	General adults	25–64	NR	13,757	3–10	Italy	Low
Therapeutic effect of dietary factors													
Tome, 2021 [46]	Vitamin D supplementation	Vitamin D vs placebo	4	10	RCTs	6 Asia, 3 EU, 1 USA	Depressed patients	NR	NR	1398 (I:696; C:702)	NA	Spain	Low
Fusar-Poli, 2021 [30]	Cocoa-rich foods	Cocoa-rich products vs placebo	2	5	RCTs	2 Asia; 1 EU; 1 USA; 1 Australia	General adults	50–80	0–100	293 (I:148; C:145)	NA	Italy	High
Yosaee, 2020 [40]	Dietary zinc supplementation	Zinc vs placebo/antidepressants	4	7	RCTs	5 Asia; 2 EU	Depressed patients	NR	NR	319 (I: 159C 160)	NA	Iran	High
Young, 2019 [48]	B vitamin supplementation	B vitamin vs placebo	4	7	RCTs	2 EU; 5 Australia	Healthy and “at risk” adults	18–69	0–100	568 (I:278C: 290)	NA	Australia	Very low
Firth, 2019 [25]	Dietary intervention	Dietary intervention vs non-dietary control	8	16	RCTs	3 USA; 4 Australia; 3 Columbia; 6 EU	Depressed patients	21–85	NR	45,958 I:1918 C:26,778	NA	United Kingdom	High
Elin, 2019 [28]	Very low-calorie diet	Very low-calorie vs control	3	11	RCTs	1 EU; 10 USA	Depressed patients	20–58	0–53	354	NR	Canada	Very low
Liu, 2019 [39]	Probiotic intervention	Probiotic vs placebo	3	23	RCTs	NR	Healthy adults	18–79	0–100	2574	NR	USA	Low
Liu, 2019 [39]	Prebiotic intervention	Prebiotic vs placebo	3	4	RCTs	NR	Depressed patients	23–54	49–51	384	NR	USA	Low
Liao, 2019 [39]	n-3 PUFA supplementation	n-3 PUFA vs placebo	2	26	RCTs	NR	Depressed patients	18–95	NR	2160 (I:1089 C:1071)	NR	China	Low
Veronese, 2018 [45]	ALC supplementation	ALC vs placebo	5	9	RCTs	EU	Depressed patients	46.3–80	20–60	467 (I:231 C:236)	NR	United Kingdom	Low
Veronese, 2018 [45]	ALC supplementation	ALC vs antidepressant	5	3	RCTs	EU	Depressed patients	45–72.2	22–33	324 (I:162 C:162)	NR	United Kingdom	Low
Sarris, 2016 [47]	Folic acid supplementation	Folic acid vs placebo	4	4	RCTs	EU	Depressed patients	>18	NR	671	NR	Australia	Very low

*AMSTAR-2* A Measurement Tool to Assess Systematic Reviews-2, *AHEI* alternate healthy eating index, *DII* dietary inflammatory index, *ALC* acetyl-l-carnitine; *n-3 PUFA* omega-3 poly-unsaturated fatty acid, *RCTs* randomized clinical trials, *NR* not reported, *NA* not appliable, *I* intervention group, *C* control group, *EU* Europe.

The definition and criteria for depression in the meta-analyses included in our umbrella review could be grouped into three categories: 8% of the studies diagnosed depression by using a structured clinical interview for the Diagnostic and Statistical Manual of Mental Disorders-IV (DSM-IV), 27% diagnosed depression by self-report physician diagnosis or anti-depression medication use, others measured depressive symptoms using a variety of questionnaires, including the Centre for Epidemiological Studies Depression Scale (CES-D), the Patient Health Questionnaire 9 (PHQ-9), and the Geriatric Depression Scale (GDS).

### The methodological quality of included meta-analyses

The results of the methodological quality assessment were shown in Supplementary Table 3. Five (17.9%), 2 (7.1%), 14 (50.0%), and 7 (25.0%) of the retrieved meta-analyses were assessed with high, moderate, low, and very low, respectively. Most of the meta-analyses had low or very low confidence of their findings because they did not adhere to the following critical domains—(1) did not mention established review protocol before conduction (20 of 21, 95.2%), and (2) did not use a satisfactory technique for assessing the risk of bias and account for it in individual studies when interpreting the results (8 of 21, 38.1%). Moreover, most meta-analyses did not report funding sources of included primary studies as well as perform study selection and data extraction in duplicate.

### Associations and quality of evidence between dietary factors and the risk of depression

**Fig. 2:**
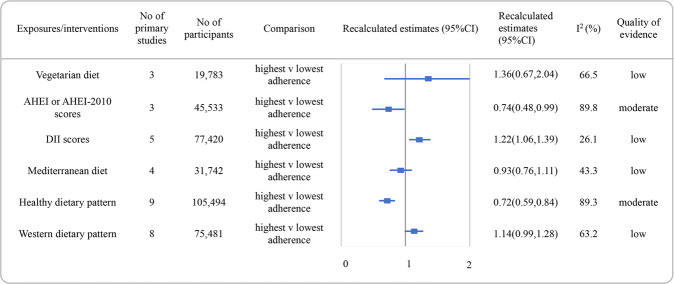
Summary estimates with 95% confidence intervals and quality of evidence for the associations between dietary patterns or dietary quality indices and the risk of depression. AHEI alternate healthy eating index, DII dietary inflammatory index, CI confidence interval.

**Fig. 3:**
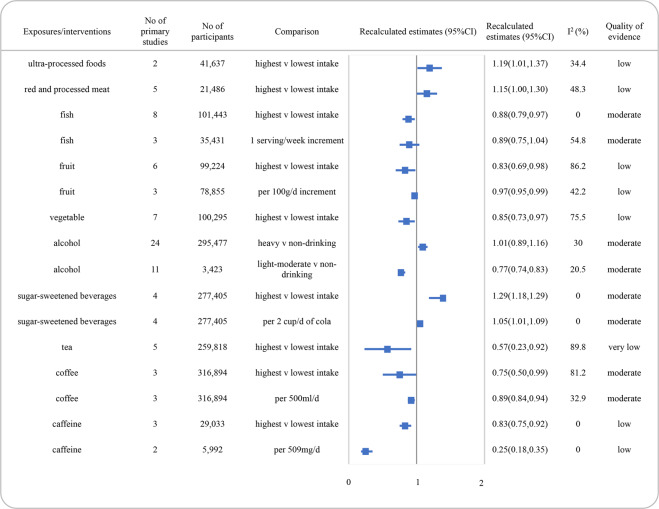
Summary estimates with 95% confidence intervals and quality of evidence for the associations between food and beverages and the risk of depression. SSBs sugar-sweetened beverages, CI confidence interval.

**Fig. 4:**
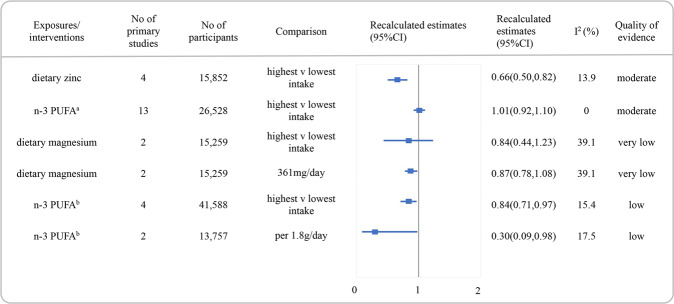
Summary estimates with 95% confidence intervals and quality of evidence for the associations between nutrients and the risk of depression. ^a^Based on meta-analysis of randomized clinical trials, ^b^based on meta-analysis of cohorts. n-3 PUFA omega-3 poly-unsaturated fatty acid, CI confidence interval.

Reanalyzed estimate with 95% CI and the quality of the evidence for each association were presented in Figs. 2, 3 and 4. The detail scores of items in NutriGrade were shown in Supplementary Table 4. Overall, none of the associations was graded as high quality of evidence. Moderate, low, and very low were evaluated for 42.9% (*n* = 12), 46.4% (*n* = 13), and 10.7% (*n* = 3) of the comparisons, respectively.

Figure 2 showed the recalculated RRs with their corresponding 95% CIs and the quality of evidence for the associations between diet quality indices or dietary patterns and the risk of depression. Higher versus lower adherence to a healthy diet had the potential to decrease the risk of depression with moderate quality of evidence (RR: 0.72, 95% CI, 0.59–0.84, *I*^2^ = 89.3%). An inverse association was found between higher AHEI or AHEI-2010 scores and depression incidence and also graded as moderate quality of evidence (RR: 0.74, 95% CI, 0.48 to 0.99, *I*^2^ = 89.8%). The associations between diet of high DII scores and increased depression risk were rated as low quality of evidence. Additionally, no association was found between the adherence to vegetarian diet, Mediterranean diet, or western diet and depression incidence, with low quality of evidence.

The summary RRs for associations between food groups and the risk of depression and quality of evidence were presented in Fig. 3. We identified an inverse association between consumption of fish for high vs low comparison and depression incidence, graded with moderate quality of evidence (RR: 0.88, 95% CI, 0.79–0.97, *I*^2^ = 0.0%). Low-quality evidence was found for the associations between increased intake of red and processed meat and ultra-processed foods and the increased risk of depression. Moreover, the inverse association between intake of fruits (per 100 g increment) or vegetables and the risk of depression was graded with low as well.

For beverages, the quality of evidence was moderate for a decreased risk of depression with light to moderate consumption of alcohol (<40 g/day, RR: 0.77, 95% CI, 0.74–0.83, *I*^2^ = 20.5%). While there was no significant association between heavy drinking on alcohol and depression risk (>48 g/day, RR: 1.01, 95% CI, 0.89–1.16, *I*^2^ = 30.0%), with moderate quality of evidence. Meanwhile, the inverse association between coffee and the risk of depression (for per 500 ml/day, RR: 0.89, 95% CI, 0.84–0.94, *I*^2^ = 32.9%), as well as the positive association between SSBs consumption and the risk of depression (for per 2 cups/day cola, RR: 1.05, 95% CI, 1.01–1.09, *I*^2^ = 0.0%) in dose-response meta-analysis, were graded with moderate quality of evidence. An inverse association between caffeine intake and depression with low quality of evidence was also found.

Figure 4 presented the RRs with their 95% CI and quality of evidence for the associations between nutrients and the risk of depression. Moderate quality of evidence was discovered for an inverse association between dietary zinc and the risk of depression (RR:0.66, 95% CI 0.50–0.82, *I*^2^ = 13.9%). We found no association between higher consumption of n-3 PUFA and the risk of depression in a meta-analysis based on RCTs (RR:1.01, 95% CI 0.92–1.10, *I*^2^ = 0.0%), rated as moderate quality of evidence. However, an inverse association between n-3 PUFA and depression incidence in the meta-analysis included prospective cohorts was rated as low quality of evidence. The quality of evidence for the inverse association between dietary magnesium and depression was rated as very low.

### Associations and quality of evidence between dietary factors and the treatment of depression

Table 2 showed summary estimates with 95% CI and the quality of evidence for each association between dietary factors and depression treatment in meta-analyses based on RCTs. We found healthy dietary intervention significantly reduced depressive symptoms with moderate quality of evidence (Hedges’s *g* = 0.28, 95% CI, 0.10–0.45, *I*^2^ = 89.4%). As for the gut microbiota modifier, a potential treatment target, moderate quality of evidence showed that probiotics yielded small but significant effects for depression (SMD = −0.31, 95%CI, −0.56 to −0.07, *I*^2^ = 48.2%), whereas prebiotics did not differ from placebo for depressive symptoms, rated as low quality of evidence. Besides, n-3 PUFA (SMD = −0.28, 95%CI, −0.47 to −0.09, *I*^2^ = 75.0%) and ALC (SMD = −1.10, 95%CI, −1.65 to −0.56, *I*^2^ = 86.0%) supplementations had remission roles on depression severity compared to placebo, both graded with moderate quality of evidence. Moderate quality of evidence also concluded that no obvious effect on vitamin D supplementation for depressive symptoms. The quality of evidence for the role of a very low-calorie diet, cocoa-rich foods, dietary zinc, total B vitamins, and single folate acid supplementation on depression treatment was low to very low.

**Table 2:** Summary estimates with 95% confidence intervals and quality of evidence for associations between dietary intervention and the treatment of depression.

Interventions	No. of studies	No. of participants	Comparison	Estimates	95% CI	*I*^2^ (%)	Quality of evidence
Dietary pattern							
Healthy diet	16	45,958	Diet intervention vs control	Hedges’s *g* = 0.28	(0.10, 0.45)	89.4	Moderate
Very-low calorie diet	11	354	Very low-calorie vs control	Hedges’s *g* = −0.73	(−1.20, −0.25)	32.2	Low
Food groups							
Cocoa-rich foods	5	293	Cocoa-rich foods vs placebo	Hedges’s *g* = −0.42	(−0.68, −0.17)	43.5	Low
Prebiotic	5	384	Prebiotic vs placebo	SMD = −0.08	(−0.30, 0.15)	56.8	Low
Probiotic	23	2574	Probiotic vs placebo	SMD = −0.31	(−0.56, −0.07)	48.2	Moderate
Nutrients							
Dietary zinc	8	319	Zinc vs placebo	SMD = −4.16	(−6.56, −1.75)	80.1	Low
Vitamin D	10	1398	Vitamin D vs placebo	SMD = −0.91	(−2.02, 0.19)	99.0	Moderate
B vitamins	7	568	B vitamin vs placebo	SMD = 0.15	(−0.01, 0.32)	0.0	Very low
Folate acid	4	671	Folate acid vs placebo	Hedges’s *g* = 0.49	(−0.31, 1.29)	93.0	Very low
n-3 PUFA	26	2160	n-3 PUFA vs placebo	SMD = −0.28	(−0.47, −0.09)	75.0	Moderate
ALC	9	467	ALC vs placebo	SMD = −1.10	(−1.65, −0.56)	86.0	Moderate
ALC	3	323	ALC vs antidepressant	SMD = 0.058	(−0.22, 0.34)	31.0	Low

*SMD* standardized mean differences, *CI* confidence interval, *ALC* acetyl-l-carnitine, *n-3 PUFA* omega-3 poly-unsaturated fatty acid.

### Publication bias

Our results indicated the potential publication bias according to Egger’s test (*P* < 0.1) for red and processed meat, tea consumption in meta-analyses comparing high versus low intake. The presence of publication bias was also indicated for very low-calorie diet intervention, ALC supplementation in meta-analyses comparing intervention with placebo. The funnel plots showed potential publication bias for 6 associations, including the high versus low adherence or intake meta-analyses for healthy dietary pattern, red and processed meat, fruit and tea, and the relation between treatment for depression and vitamin D and dietary zinc intervention (Supplementary Table 5)

## Discussion

### Main findings

The present umbrella review provided a broad overview of the influence of dietary patterns, food and beverages, and nutrients on the risk of depression as well as the reduction of depressive symptoms. To our knowledge, it is the first to summarize the current meta-analyses, assess the methodological quality of these publications, and evaluate the quality of evidence for all the associations on this topic.

Twenty-eight meta-analyses comprising 40 summary estimates for the associations between different dietary factors and depression were identified. None of high-quality evidence was found. Moderate quality of evidence was found for the inverse associations between healthy diet, diet of higher AHEI or AHEI-2010 scores, fish, coffee, light to moderate alcohol (<40 g/d), dietary zinc, and the risk of depression, as well as for the effect of healthy diet intervention, probiotics, n-3 PUFA and ALC intervention on depressive symptoms reduction. Besides, there was also moderate quality of evidence that high consumption of SSBs increased the risk of depression. The quality of evidence of all the remaining associations between dietary factors and depression was rated as low or very low, thus the overall summary estimates could likely to be changed in further research. In addition, the methodological quality was low or very low for most of the published meta-analyses.

### Comparison with other studies and possible explanations

#### Dietary factors and the risk of depression

The consistency of evidence supports the protective relationship between a healthy diet or diet with higher AHEI or AHEI-2010 scores and depression, and the positive association between pro-inflammatory diet and depression in our umbrella review. These findings are in agreement with published guidelines and reviews [17, 49–51]. A healthy diet shares a diet with a high intake of fruits and vegetables, fish, legumes, nuts, and cereals, with a low intake of red and processed meat, and the opposite is true for a pro-inflammatory diet [52]. Recent investigations have suggested that low-grade chronic inflammation, oxidation stress, or defective antioxidant defenses may contribute to develop psychiatric disorders, including depression [53–55]. Fruits and vegetables are rich resources of fiber, minerals, phytochemicals and contain a high level of antioxidants. Increased absorption of them can reduce oxidative stress, reducing the risk of depression [56, 57]. Our results support the inverse associations of fruits and vegetables intake with the risk of depression in high versus low consumption meta-analyses, with low quality of evidence. A lower level of brain-derived neurotrophic factor (BDNF) and abnormality in neuroplasticity or cognitive ability which are involved in the pathogenesis of depression were proven to be connected with a high level of saturated fats in red and processed meat [3, 58]. Our overview also found a harmful relationship of the risk of depression with a higher intake of red and processed meat. Besides, fish and nuts contain high levels of n-3 PUFA, which has the potential to prevent depression through anti-inflammatory effect, neuro-endocrine modulation, and neurotransmitter activation [59–61]. The inverse association between fish or n-3 PUFA and depression incidence, with moderate or low quality of evidence, respectively, was also found in this umbrella review. Furthermore, the meta-analysis based on RCTs showed inconsistent conclusions on the effect of n-3 PUFA on depression incidence, partly due to their short-term trials (no more than 12 months), limited dosages or variations on n-3 PUFA sources, and so on. Thus, further research in this area to investigate the direction of this association is needed.

There was moderate-quality evidence for negative effect of SSBs on the risk of depression that was identified in our umbrella review. The Seguimiento Universidad de Navarra (SUN) project from 1999 through 2016 among 15,546 Spanish university graduates with 769 depression cases found that the highest intake of added sugar consumption was associated with increased depression risk [62]. Further study from the Whitehall II study also found that sugar from food and beverages was related to higher depression incidence, prevalence, and recurrence of mood disorders, including depression [63]. Evidence from animal studies has turned out that a diet rich in sugar during peri-adolescence increases depressive-like behavior in their adulthood via activating the hypothalamic-pituitary-adrenal (HPA) axis and inducing elevation in glucocorticoids [64]. As the largest consumer of SSBs, overconsumption of added sugar during adolescence also is likely to promote long-term dysregulation of the stress response [65]. In addition, SSBs are partly responsible for obesity and may predict impaired glucose homeostasis and insulin resistance leading to type II diabetes [66]. And a bi-directional relationship between obesity or type II diabetes and depression has been reported in prospective research [67, 68].

Alcohol has been reported to associate with depression. Previous cross-sectional studies concluded that problem drinkers were more vulnerable to develop depression [69, 70]. But individuals with depression were more likely to have alcohol misuse to relieve their distress, which might lead to an overestimation of the effect of alcohol on the risk of depression. In our umbrella review, we included the meta-analysis of longitudinal studies to avoid bias and found that more than 48 g alcohol intake per day did not increase depression incidence, with moderate quality of evidence. Moderate quality of evidence was also identified the inverse association between less than 40 g alcohol intake per day and the risk of depression. This is consistent with a previous genome-wide analysis, which reported the hazardous consequences related to alcohol were more genetically associated with depression than alcohol intake itself [71].

A high intake of coffee was associated with a significant reduction in the risk of depression in our study. Recent evidence suggested that coffee had several potential effects on health, including the prevention of cardiovascular risk factors, metabolic diseases, cancer, and depression [72–76]. However, the mechanism behind the preventive effect on depression are unknown. There are some hypothetical biological explanations. As an excellent source of caffeine, coffee has the potential to stimulate the central nervous system and enhance dopaminergic neurotransmission [77]. Our findings also support this hypothesis to a certain extent, because caffeine has been shown to reduce the risk of depression with low quality of evidence. Beyond caffeine, several compounds in coffee may play a role in preventing depression. For instance, chlorogenic acid, catechol, trigonelline, and *N*-methylpyridinium contained in coffee have been proven to counteract the depressed status via increasing calcium signaling and dopamine release [78]. Nevertheless, tea that is rich in caffeine and phytochemicals is not associated with the risk of depression, more research is needed to explore this field in depth.

#### Dietary factors as treatments for depression

Guidelines suggest a combination of psychological and pharmacological therapies to treat depression [79, 80]. Currently, up to 60% of the patients with depression experience some degree of nonresponse to pharmacological treatment, because of delayed onset of effect by targeting neurotransmitter activity [81]. Meanwhile, incomplete compliance with antidepressants is also frequent, mostly owing to their adverse side-effect [82, 83]. Consequently, research exploring novel treatment approaches is growing, and nutritional psychiatry becomes a newly emerging field aiming at nutritional prevention and treatment strategies for psychosomatic diseases like depression [84]. In this umbrella review, we identified the therapeutic effect of n-3 PUFA, ALC, and probiotic supplementations on depressive symptoms graded with moderate quality of evidence, as well as for dietary zinc on depression treatment rated as low quality of evidence.

Long-chain n-3 PUFA, especially docosahexaenoic acid (DHA) and eicosapentaenoic acid (EPA) [85], has been considered to be used as an adjuvant treatment for major depression thanks to their anti-inflammatory activity and maintenance of membrane integrity and fluidity [86, 87]. However, we could not draw a conclusion on the optimal dosage of DHA or EPA contributing to improvements of depression, limited by heterogeneity between primary studies in this study. A recent pairwise and network meta-analysis found that a high dose of n-3 PUFA (<2000 mg/d) might be superior to a low dose of n-3 PUFA in the early stage of major depressive disorder [88]. It was found that EPA combined with DHA therapy had significantly reduced the severity of depressive symptoms compared to the DHA monotherapy [89]. Furthermore, proper proportion on DHA with EPA is another key consideration in current studies. Song et al. found that 2:1 or 3:1 of EPA to DHA would be the most effective for depression [90]. Similarly in the other two meta-analyses, the effective ratio of EPA in treating depression was EPA ≥ 80 or ≥60%, respectively [91, 92]. These proportions may lie in the fact that EPA has a better antidepressant effect. In contrast to DHA, EPA can rapidly enter the brain and quickly act as an effector [85]. EPA and DHA can act as natural ligands of peroxisome proliferator-activated receptor γ (PPARγ) and inhibit the neuronal para-inflammatory cascade in the pathophysiological process of depression [93]. DHA binding depends on concentration, while a low concentration of EPA could bind with very high affinity to all PPARs [94]. Even so, we could not determine the efficacy occurs because of EPA alone or because of an interaction with DHA supplementation, and the optimal proportion or dosage of both DHA and EPA supplementation in improving depressive symptoms. Therefore, future studies should deeply explore the anti-depression effect of DHA and EPA from the aspects such as different populations and appropriate dosage.

Recently, complex bi-directional communication between the gut and the brain has been identified to play an important role in the pathology of depression [95, 96]. Probiotics, as gut microbiota modifiers, hold particular appeal partly for depression treatment. Marcos et al conducted the first study to explore the relief efficacy of probiotics on depressive symptoms among healthy students in 2004 [97]. During the last decades, a growing number of studies have confirmed the anti-depression properties of probiotics in healthy populations [98, 99]. Yet there is limited evidence from clinical trials of clinical depressed populations. Our findings indicated a beneficial association between probiotics supplementation and the treatment of depression among depressed patients, but with a high risk of methodology bias and moderate quality of evidence. The number of RCTs in this overview was limited, and there was a large gap among the primary trials on the characteristic of participants, type of intervention, intervention duration, and combination of probiotics strains, which reduced the capacity to draw a clinically meaningful conclusion. There is an urgent need to determine the exact effect of this novel treatment approach, and to investigate potential underlying mechanisms. Further well-designed studies should: (1) consider differences in microbiome composition across the lifespan; (2) explore specific probiotics combination according to all ages of patients with depression; (3) take inter-individual variation into account.

Carnitine, widely known for its function on peripheral lipid metabolism, has been reported to take part in brain lipids synthesis and improve neurofunction via increasing antioxidant activity and enhancing cholinergic neurotransmission [100]. Of particular interest, ALC has been recognized as an effective treatment for geriatric depression, which is associated with the normalization of phosphomonoester levels in the prefrontal region [101, 102]. A recent meta-analysis found that ALC was effective and tolerable for general adults with depression [103]. Our findings confirmed the therapeutic effect of ALC among general depressed adults. Future studies with a larger sample size could be of importance to examine whether ALC is applicable in the general population without inacceptable adverse effects.

Micronutrients have been deemed to be the most prominent and valid substitutes for a monoamine-based antidepressant. Several studies have concluded that zinc deprivation could induce depressive-like behavior, while zinc supplementation could reverse the situation effectively [104, 105]. Similarly, findings from this umbrella review showed mood-improving properties of zinc supplementation among patients with depression, evaluated with low quality of evidence. However, we still need to be cautious with the conclusion. First, it was difficult to distinguish the true effect of zinc supplementation on the severity of depression on account of variations in criteria of depression grades in primary studies. Additionally, whether zinc supplementation has sustainable effects remains unknown, which provides a feasible avenue for further research.

### Strengths and limitations

Our umbrella review had several strengths. It was the first broad overview of the meta-analyses on the association of any dietary factors and the prevention and treatment of depression. We had taken measures to minimize bias in the umbrella review, e.g., recalculating all meta-analyses using a random effect method, conducting the review process by two authors independently. Furthermore, we also evaluated the methodological quality and the quality of evidence for all identified associations by using validated tools. By uncovering research gaps, we could identify relevant future research directions.

This umbrella review also had some limitations. First, we did not include qualitative systematic reviews. Second, confounding was the major concern in meta-analyses based on observational studies. Although the most important confounders were adjusted for in most of the primary studies (82% for age and sex, 78% for smoking, 70% for educational level, and 65% for BMI and total energy intake), the residual confounders could not be completely avoided. For instance, only 46% of the studies adjusted for alcohol intake, which should be considered in further studies. Third, some new individual primary studies and primary studies that were not included in any published meta-analyses might have been missing, as well as some outcomes without meta-analysis were not summarized. Finally, owing to the limited studies, we did not conduct subgroup analysis (e.g., exploring by age, sex, geographical location), or sensitive analysis (e.g., excluding studies with high risks), and other relevant factors might have been missed. For example, regarding dietary zinc intake and the incidence of depression, evidence showed the difference between US, Asian, and European populations, with a decreased incidence of depression in Asian and US populations, and no association for European countries [106].

### Conclusions and future directions

The present umbrella review provided a comprehensive overview of the currently available meta-analyses focusing on the relationship between dietary factors and the prevention and treatment of depression. There was moderate-quality evidence for the inverse associations between adherence to a healthy diet, high AHEI or AHEI-2010 diet scores, fish, coffee, light to moderate alcohol intake, and dietary zinc intake, and the risk of depression. In addition, several meta-analyses were identified for the positive association between SSBs intake and the risk of depression, assessed as the moderate quality of evidence. Moderate quality of evidence had been found of the therapeutic effects of probiotics, n-3 PUFA, and ALC supplementation on depression as well.

To achieve high-quality evidence for the impact of dietary factors on depression, and be able to draw strong conclusions, future studies should pay attention to several aspects. It should be foremost to improve dietary measurement tools and get dietary data with high validity in observational studies. Depression assessment is another concern of the investigation in the preventive effect of dietary factors for depression. DSM-IV applied by experienced psychiatrists should be considered in research as the “gold standard”. Although questionnaires have high validity for depression diagnosis, the end-points on the same scale are inconsistent among studies. Further exploration on standardizing the application of these questionnaires to control heterogeneities is needed. Moreover, studies should investigate exposures that have biological potential effects on depression, but for which no summary evidence is available, or the current quality of evidence is low. We found no meta-analysis examining the association between intake of whole-grain/cereals, nuts, legumes, dairy products, dietary calcium, or dietary iron and the risk of depression, which could have a potential effect on depression. More studies are also needed on specific food or nutrients, such as prebiotics, tea, n-3 PUFA, vitamin D, and B vitamins. Considering the methodological quality, authors should be more careful in the synthesis of primary studies, for instance, to avoid including different study designs, pooling different exposures or outcomes, and double calculating the same population. Sufficient assessments of the risk of bias of included studies using valid tools are needed for future meta-analyses. If possible, conducting linear or nonlinear dose-response meta-analysis would be more persuasive. Meanwhile, it is highly recommended for authors to register a protocol (e.g., PROSPERO) prior to conduction, use a satisfactory technique for assessing and accounting bias, and follow standardized guidelines such as the PRISMA, to ensure high methodological quality.

## Supplementary information

Supplementary table 1

Supplementary table 2

Supplementary table 3

Supplementary table 4

Supplementary table 5
