# Supplementary material for: Role of dietary factors in the prevention and treatment for depression: an umbrella review of meta-analyses of prospective studies
Source: Transl Psychiatry. 2021 Sep 16;11:478. doi: 10.1038/s41398-021-01590-6 (PMC8445939; doi:10.1038/s41398-021-01590-6)
Supplement: Supplementary file 1 — Supplementary table 1 [file 41398_2021_1590_MOESM1_ESM.pdf]

Supplementary table 1. Search terms and search strategies from PubMed, Embase, and Cochrane Library

**PubMed**	
#1	((((((((((Depression*[Title/Abstract]) OR (Depressive Symptom*[Title/Abstract])) OR (Emotional Depression*[Title/Abstract])) OR (Depressive Disorder*[Title/Abstract])) OR (Depressive Syndrome*[Title/Abstract])) OR (Melancholia*[Title/Abstract])) OR (Unipolar Depression*[Title/Abstract])) OR (Postnatal Depression*[Title/Abstract])) OR (Post-Partum Depression*[Title/Abstract])) OR (Major Depressive Disorder*[Title/Abstract])) OR (Post-Partum Depression*[Title/Abstract])) OR (Post-Natal Depression*[Title/Abstract])) OR (("Depression"[Mesh] OR "Depressive Disorder"[Mesh] OR "Depression, Postpartum"[Mesh]) OR "Depressive Disorder, Major"[Mesh])
#2	((((((("Diet"[Mesh]) OR "Food"[Mesh]) OR "Beverages"[Mesh]) OR "Fermented Foods and Beverages"[Mesh]) OR "Dietary Supplements"[Mesh]) OR "Micronutrients"[Mesh]) OR "Minerals"[Mesh]) OR "Polyphenols"[Mesh]) OR (((((((((((((((((((diet*[Title/Abstract]) OR (dietary[Title/Abstract])) OR (nutrition[Title/Abstract])) OR (food*[Title/Abstract])) OR (nutrient*[Title/Abstract])) OR (drink*[Title/Abstract])) OR (beverage*[Title/Abstract])) OR (supplement*[Title/Abstract])) OR (supplementation[Title/Abstract])) OR (intake[Title/Abstract])) OR (eating[Title/Abstract])) OR (drinking[Title/Abstract])) OR (consumption[Title/Abstract])) OR (macronutrient*[Title/Abstract])) OR (micronutrient*[Title/Abstract])) OR (trace element*[Title/Abstract])) OR (phytochemical*[Title/Abstract])) OR (polyphenol*[Title/Abstract])) OR (vitamin*[Title/Abstract])) OR (mineral*[Title/Abstract]))
#3	((((((systematic review*[Title/Abstract]) OR (comprehensive review*[Title/Abstract])) OR (systematic overview*[Title/Abstract])) OR

	(comprehensive overview*[Title/Abstract])) OR (meta-analys*[Title/Abstract])) OR (metaanalys*[Title/Abstract])) OR (("Review Literature as Topic"[Mesh]) OR "Meta-Analysis as Topic"[Mesh])
#4	#1 AND #2 AND #3
**Embase**	
#1	postnatal depression/ or depression/ or major depression/ or postoperative depression / or perinatal depression/
#2	(depression* or depressive symptom* or emotional depression* or depressive disorder* or depressive syndrome* or melancholia* or unipolar depression* or postnatal depression* or post-partum depression* or major depressive disorder* or postpartum depression* or post-natal depression*).mp.
#3	#1 or #2
#4	Food/ or diet/ or beverage/ or fermented product/ or dietary supplement/ or trace element/ or mineral/ or polyphenol/
#5	(diet* or dietary or nutrition or food* or drink* or beverage* or supplement* or supplementation or intake or eating or drinking or consumption or macronutrient* or micronutrient* or macronutrient* or trace element* or photochemical* or polyphenol* or mineral* or vitamin*).mp.
#6	#4 or #5
#7	“systematic review”/ or meta analysis/
#8	(“systematic review*” or “comprehensive review*” or “systematic overview*” or “comprehensive overview*” or meta-analys* or metaanalys*).mp.
#9	#7 or #8
#10	#3 AND #6 AND #9
**Cochrane Library**	
#1	([Diet] explode all trees) OR ([Food] explode all trees) OR ([Beverages] explode all trees) OR ([Fermented Foods and Beverages] explode all trees) OR ([Dietary Supplements] explode all trees) OR ([Micronutrients] explode all trees) OR

	([Minerals] explode all trees) OR ([Polyphenols] explode all trees)
#2	(diet*):ti,ab,kw OR (dietary):ti,ab,kw OR (nutrition):ti,ab,kw OR (food*):ti,ab,kw OR (nutrient*):ti,ab,kw OR (drink*):ti,ab,kw OR (beverage*):ti,ab,kw OR (supplement*):ti,ab,kw OR (supplementation):ti,ab,kw OR (intake):ti,ab,kw OR (eating):ti,ab,kw OR (drinking):ti,ab,kw OR (consumption):ti,ab,kw OR (macronutrient*):ti,ab,kw OR (micronutrient*):ti,ab,kw OR (trace element*):ti,ab,kw OR (phytochemical*):ti,ab,kw OR (polyphenol*):ti,ab,kw OR (vitamin*):ti,ab,kw OR (mineral*):ti,ab,kw
#3	#1 OR #2
#4	([Depression] explode all trees) OR ([Depressive Disorder] explode all trees) OR ([Depression, Postpartum] explode all trees) OR ([Depressive Disorder, Major] explode all trees)
#5	(depression*):ti,ab,kw OR (depressive symptom*):ti,ab,kw OR (depressive disorder*):ti,ab,kw OR (depressive syndrome*):ti,ab,kw OR (melancholia*):ti,ab,kw OR (unipolar depression*):ti,ab,kw OR (postnatal depression*):ti,ab,kw OR (post-partum depression*):ti,ab,kw OR (major depressive disorder*):ti,ab,kw OR (post partum depression*):ti,ab,kw
#6	#4 OR #5
#7	([Systematic Reviews as Topic] explode all trees) OR ([Meta-Analysis as Topic] explode all trees)
#8	(systematic review*):ti,ab,kw OR (systematic review*):ti,ab,kw OR (systematic overview*):ti,ab,kw OR (comprehensive overview*):ti,ab,kw OR (meta-analys*):ti,ab,kw
#9	#7 OR #8
#10	#3 AND #6 AND #9 in Cochrane Reviews, Cochrane Protocols
