# Supplementary material for: Role of dietary factors in the prevention and treatment for depression: an umbrella review of meta-analyses of prospective studies
Source: Transl Psychiatry. 2021 Sep 16;11:478. doi: 10.1038/s41398-021-01590-6 (PMC8445939; doi:10.1038/s41398-021-01590-6)
Supplement: Supplementary file 2 — Supplementary table 2 [file 41398_2021_1590_MOESM2_ESM.pdf]

Supplementary Table 2. Detail characteristics of included meta-analyses in this umbrella review.

PMID	Author (year)	Dietary intervention/exposures	Comparion	Database searched	Search period	No of studies	Type of included studies	Settings	Participants	Ages	%Male	Total N	Follow years	Funding	Country of author	Recalculated ES with 95%CI	I^2	ASMART-2
32792031	Pagliai, 2021	ultra-processed foods	highest vs lowest intake	5(MEDLINE, Embase, Scopus, Web of Science and Google Scholar databases,)	to June 2020	2	Cohort	2EU	general adults	18-86	NR	41637	NR	None	Italy	1.19(1.01,1.37)	34.4%	high
33533015	Tome, 2021	vitamin D supplementation	vitamin D vs placebo	4(PubMed, Embase, Web of Science-Science Citation Index)	to September 21 2019	10	RCTs	6 Asia, 3 EU, 1 USA	depressed adult patients	NR	NR	1398 (I:696; C:702)	NR	NR	Spain	-0.91(-2.02,0.19)	99.0%	low
33809274	Markun ,2021	B vitamin supplementation	B vitamin vs placebo	5(MEDLINE® (via Ovid), Embase®, PsycINFO, Cochrane Library, and Scopus）	in June 2020	5	RCTs	2 Australia; 2 EU; 1USA	depressed adult patients	＞65	0-50	5158	NR	None	Switzerland	-0.03(-0.12, 0.06)	15.1%	high
34026809	Chen,2021	DII	higherst adherence vs lowest adherence	3(PubMed, Embase, and Web of Science)	to December 31,2020	4	Cohort	2EU; 1Australia; 1USA	general adults	18-69	0-42	28662(4083cases)	8.5-12.6	Institution	China	1.28(1.17,1.39)	39.5%	low
33970709	Fusar-Poli, 2021	cocoa-rich foods	cocoa-rich products vs placebo	5(Web of Science, MEDLINE, KCI – Korean Journal Database, Russian Science Citation Index, and SciELO Citation Index),	to April 3,2020	5	RCTs	2 Asia; 1 EU; 1 USA; 1 Australia	depressed adult patients	50-80	0-100	293 (I:148; C:145)	NR	None	Italy	-0.422(-0.675,-0.169)	43.5%	high
31837230	Li,2020	alcohol intake	heavy drinking（＞48g/d) vs non-drinkers	3(PubMed ,Embase,PsycINFO	to 15 April 2019	24	Cohort	12 EU; 6 USA; 5 Australia; 1 Canada	general adults	18-99	0-100	295477 (21378 cases)	1-40	Government	China	1.01(0.89,1.15)	40.5%	low
29076953	Veronese,2018	Acetyl-L-Carnitine supplementation intervention	ALC vs placebo	5(PubMed, EMBASE, SCOPUS, Cochrane Central Register of Controlled Trials, and Clinicaltrials.gov)	until December 30, 2016	9	RCTs	7 Italy; 1Russian; 1 Norway	depressed adult patients	46.3-80	20-60	467(intervention 231;control 236		None	United Kindom	-1.1(-1.65,-0.65)	86.0%	low
30254236	Lassale, 2019	AHEI or AHEI-2010	highest adherence vs lowest adherence	3(Medline, Embase and PsychINFO	to 31st May 2018	3	Cohort	EU	general adults	37-61	NR	45533(3477 cases）	5-8.5	Fundation	United Kindom	0.74(0.48,0.99)	80.7%	moderate
31837230	Li,2020	alcohol intake	light-moderate drinking （＜40g/d) vs non-drinkers	3(PubMed ,Embase,PsycINFO	to 15 April 2019	11	Cohort	NR	general adults	18-99	0-100	3423 cases	1-40	Government	China	0.81(0.75,0.87)	43.6%	low
26339067	Wang,2016	caffeine exposure	highest intake vs lowest intake	3(PubMed database, Web of Science China National Knowledge Infrastructure (CNKI) and WANFANG DATA"	up to 1 May 2015	4	Cohort	3 EU; 1 USA	general adults	>18	NR	29033	NR	Fundation	China	0.83(0.75,0.92)	0.0%	very low
26339067	Wang,2016	caffeine exposure	509mg/day	3(PubMed database, Web of Science China National Knowledge Infrastructure (CNKI) and WANFANG DATA"	up to 1 May 2015	1	Cohort	3 EU; 1 USA	general adults	>18	NR	5992(669 cases	NR	Fundation	China	0.25(0.18,0.35）	not appliable	very low

29076953	Veronese,2018	Acetyl-L-Carnitine supplementation intervention	ALC vs antidepressant	5(PubMed, EMBASE, SCOPUS, Cochrane Central Register of Controlled Trials, and Clinicaltrials.gov)	until December 30, 2016	3	RCTs	3 Italy	depressed adult patients	45-72.2	22-33	324(intervention 162; control 162		None	United Kindom	0.06(-0.22,-0.34）	31.0%	low
29804517	Wu,2020	AHEI-2010 scores or AHEI scores	highest adherence vs lowest adherence	9(Google Scholar, PubMed, Scopus, Web of Science, Elsevier Word mark, Cochrane library, Clinical Trial, Airiti Library, and the National Digital Library of Theses and Dissertations in Taiwan	up to December 2016	2	cohort	EU	general adults	NR	NR	19308(1311 cases）	10-15	None	China	0.57(0.47,0.67)	0.0%	low
31527485	Young, 2019	B vitamin supplementation	B vitamin vs placebo	4(PubMed andWeb of Science, Scopus, and PsycINFO	until January 2019	7	RCTs	NR	healthy and “ at risk” adults	18-69	0-100	568 (intervention 278 control 290)		Institution	Australia	0.15(-0.01,0.32）	0.0%	very low
29031185	Molendijk,2018	healthy dietary pattern	highest adherence vs lowest adherence	3(Embase,PUBMED, and Web of Science	up to March 6th, 2017	9	Cohort	3 EU; 2 Australia; 1 USA; 2 Canada;1 Asian	general adults	18-74	0-75	105494	6.5 month-12.7	NR	Netherlands	0.77 (0.67,0.89	87.7%	moderate
24196402	Lai,2014	healthy dietary pattern	highest adherence vs lowest adherence	5(Medline, Embase, and PsycInfo via Ovid,EBSCO,Proquest	up to August 2013	4	Cohort	2 EU;2 USA	general adult population	18-85	0-57.6	68416	4.4-15	None	Australia	0.77(0.57,0.98)	94.0%	low
30720698	Firth,2019	dietary intervention	dietary intervention vs non dietary control	6(OVIDMedline,Cochrane Central Register of Controlled Trials, Health Technology Assessment Database, Allied and Complementary Medicine (AMED), Embase, Health Management Information Consortium (HMIC), and PsycINFO	December 03, 2018	16	RCTs	NR	depressed adult patients	21-85	NR	45958(intervention 19180; control 26778		institution	United Kindom	0.275(0.1,0.45)	89.4%	high
32829928	Yosaee, 2020	dietary zinc supplementation	zinc vs placebo/antidepressants	4 (PubMed,EMBASE, Scopus, and ISI web of science)	up to 14 May2020	7	RCTs	5 Iran; 2 Poland	depressed adult patients	NR	NR	319 (intervention 159; control 160)		NR	Iran	-4.15(-6.56, -1.75)	80.1%	high
32885249	da Silva,2020	dietary zinc supplementation	zinc vs placebo/antidepressants	5("MEDLINE/PubMed, LILACS, CENTRAL，Web of Science, and Embase databases)	until September 2019	5	RCTs	3 Poland 2 Iran	depressed adult patients	18-80	NR	192(intervention 96； control 96)		None	Brazil	-0.36(-0.67,-0.04)	70.6%	low
27113121	Sarris,2016	folic acid supplementation	folic acid vs placebo	4(PubMed, CINAHL, Web of Science, and Cochrane Library	up to December 2015	4	RCTs	NR	depressed adult patients	＞18	NR	671		NR	Australia	0.487(-0.314,1.288）	93.0%	very low

28431261	Li Y,2017	healthy dietary pattern exposure	highest adherence vs lowest adherence	2(MEDLINE and EMBASE databases	up to September 2016	12	Cohort	5 EU;3 Asia; 2 Australia; 1 Canada; 1 USA	general adults	20-77	NR	103076	NR	Government	China	0.68(0.59,0.77)	79.0%	low
30319085	Wang,2018	inflammatory potential of the diet	highest adherence vs lowest adherence	3(PubMed, Web of Science and EMBASE databases	to August 2018	4	Cohort	2 EU;1 USA; 1 Australia	general adults	26-70	0-57.7	28662(3216 cases)	8-12.6	Government	China	1.25(1.12,1.40)	0.0%	low
30254236	Lassale, 2019	Inflammatory potential of the diet (DII)	lowest adherence vs highest adherence	3(Medline, Embase and PsychINFO	to 31st May 2018	5	Cohort	3 EU; 1 USA; 1 Australia	general adults	37-61	NR	32908(3481 cases)	5-12.6	Fundation	United Kindom	0.70(0.59,0.82)	55.3%	moderate
26518745	Grosso, 2016(2)	caffeine exposure	highest intake vs lowest intake	2(PubMed Embase	up to June 2015	3	Cohort	2 EU; 1 USA	general adults	NR	NR	58756(2656 cases		NR	Italy	0.84(0.75,0.92)	0.0%	low
26518745	Grosso, 2016(2)	caffeine exposure	100ml/d increment	2(PubMed Embase	up to June 2015	3	Cohort	2 EU; 1 USA	general adults	NR	NR	58756(2656 cases		NR	Italy	0.97(0.95,0.99)	0.0%	low
31383846	Liao,2019	n-3 fatty acid intervention	n-3 fatty acid vs placebo	2("PubMed,the Ovid version of EMBASE	to 20 December 2017	26	RCTs	NR	depressed adult patients	NR	18-95	2160 (intervention 1089, placebo 1071)		Government	China	-0.28(-0.47,-0.09)	75.0%	low
30138808	Bai,2018	n-3 fatty acid intervention	n-3 vs placeBo	9("MEDLINE, EMBASE, Web of Science, Cochrane Library, PsycINFO, Global Health, CINAHL, China National Knowledge Infrastructure (CNKI), ClinicalTrials.gov, and Chinese Biomedical Medicine Database"	to June 4, 2018	9	RCTs	6 EU; 2 Asian; 1Australia	depressed adult patients	70-90	NR	3549(intervention 1821; control 1728		institution	China	-0.202(-0.463,0.06	NR	very low
30502975	Tolkien,2019	inflammatory potential of the diet（DII or cytokine）	higherstadherence vs lowest adherence	2(PubMed and Scopus)	to 3rd October 2018	7	Cohort	4 EU; 2 USA; 1 Australia	general adults	26-81	0-75	77420	5-12.6	None	United Kindom	1.23(1.08,1.39)	40.8%	low
29500461	Kang,2018	coffee exposure	highest intake vs lowest intake	2(PubMed and Web of Science	to February 2017	3	cohort	2 USA; 1 EU	general adults	50-71	0-100	305583(13967 cases	NR	Government	South Korea	0.76(0.55,0.98)	58.8%	very low
26339067	Wang,2016	coffee exposure	highest intake vs lowest intake	3(PubMed database, Web of Science China National Knowledge Infrastructure (CNKI) and WANFANG DATA"	up to 1 May 2015	2	Cohort	1 EU; 1 USA	general adults	>18	NR	52971	NR	Foundation	China	0.57(0.02,1.12)	80.4%	very low
26339067	Wang,2016	coffee exposure	each cup/d increment	3(PubMed database, Web of Science China National Knowledge Infrastructure (CNKI) and WANFANG DATA"	up to 1 May 2015	2	Cohort	1 EU; 1 USA	general adults	>18	NR	52971	NR	Fundation	China	0.92(0.87,0.97)	not appliable	very low
26518745	Grosso, 2016(2)	coffee exposure	highest intake vs lowest intake	2(PubMed Embase	up to June 2015	3	Cohort	2 USA; 1 EU	general adults	NR	NR	316894(4656 cases		NR	Italy	0.74(0.5,0.99)	81.6%	low

26518745	Grosso, 2016(2)	coffee exposure	500ml/d increment	2(PubMed Embase	up to June 2015	3	Cohort	2 USA; 1 EU	general adults	NR	NR	316894(4656 cases		NR	Italy	0.92(0.87,0.97)	32.9%	low
30726966	Shafiei, 2019	Mediterranean diet	highest adherence vs lowest adherence	5(PubMed/MEDLINE, ISI Web of Science, Scopus,Embase, and Google Scholar"	to May 2018	4	Cohort	2 EU;1 Australia;1 USA	general adults	49.5-69	NR	31742	8-12.6	Institution	Iran	0.93(0.76,1.11)	43.3%	low
29540267	Bae,2018	n-3 fatty acid intervention	n-3 vs placeBo	5(Ovid-MEDLINE ; EMBASE ; Web of Science ; Cochrane Library ; and Korean databases	to September 2016	6	RCTs	4 EU; 1 Asian; 1 Australia	depressed adult patients older	65-80	NR	4605		NR	South Korea	-0.51(-0.98,-0.04)	84.1%	low
27113121	Sarris,2016	n-3 fatty acid intervention	n-3 vs placeco or EPA vs DHA	4(PubMed, CINAHL, Web of Science, and Cochrane Library	up to December 2015	11	RCTs	NR	depressed adult patients	＞18	NR	448		NR	Australia	0.61(0.15,1.06)	82.0%	very low
27807012	Li BR,2017	dietary magnium exposure	highest intake vs lowest intake	8(MEDLINE, Web of Science, Embase, Cochrane CENTRAL, Cumulative Index to Nursing and Allied Health Literature (CINAHL) database, CNKI, Wan fang databases and VIP	to September 2016,	2	Cohort	EU	general adults	37-62	NR	15259	6.3-20	None	China	0.84(0.44,1.23)	39.1%	very low
27807012	Li BR,2017	dietary magnium exposure	361mg/day	8(MEDLINE, Web of Science, Embase, Cochrane CENTRAL, Cumulative Index to Nursing and Allied Health Literature (CINAHL) database, CNKI, Wan fang databases and VIP	to September 2016,	2	Cohort	EU	general adults	37-62	NR	15259	6.3-21	None	China	0.87(0.78,0.98)	Not appliable	very low
32829928	Yosaee, 2020	dietary zinc exposure	highest intake vs lowest intake	4 (PubMed,EMBASE, Scopus, and ISI web of science)	up to 14 May2020	4	cohort	3 Austrialia;1 Finland	general adults	＞54.7	NR	15852(2243 cases)	3-20	Institution	Iran	0.66(0.50,0.82)	13.9%	high
28189077	Li ZY,2017	dietary zinc exposure	highest intake vs lowest intake	5(PubMed, Embase, Web of Science, Chinese National Knowledge Infrastructure (CNKI) and Wanfang databases	up to January 2017	3	Cohort	2 Australia; 1 EU	general adults	42-85	0-100	14147(2160 cases)		None	China	0.72(0.58,0.87)	0.0%	very low
26978738	Mocking,2016	n-3 fatty acid intervention	n-3 vs placeco	2(Medline and Embase databases	to 8 December 2015	13	RCTs	NR	depressed adult patients	35-60	NR	1233		institution	Netherlands	0.398(0.114, 0.682）	73.4%	very low
30254236	Lassale, 2019	Mediterranean diet	highest adherence vs lowest adherence	3(Medline, Embase and PsychINFO	to 31st May 2018	4	Cohort	2 EU; 2 Australia	general adults	37-69	NR	36556(1954 cases)	8.5-12.6	Fundation	United Kindom	0.64(0.5,0.78)	33.1%	moderate
29031185	Molendijk,2018	Mediterranean diet	highest adherence vs lowest adherence	3(Embase,PUBMED, and Web of Science	up to March 6th, 2017	5	Cohort	2 EU; 2 Australia; 1 USA	general adults	30-71	0-42	38,366	6.5 month-12.6	NR	Netherlands	0.75 (0.67,0.84)	66.0%	moderate
29031185	Molendijk,2018	unhealthy dietary pattern	highest adherence vs lowest adherence	3(Embase,PUBMED, and Web of Science	up to March 6th, 2017	9	Cohort	4 EU; 2 USA;1 Asia; 1 Australia; 1 Canada	general adults	20-74	0-73	84870	6.5 month-12	NR	Netherlands	1.05 (0.99,1.12)	45.2%	moderate

32885996	Askari, 2020	vegetarian diet	highest adherence vs lowest adherence	3(Scopus, PubMed, and Web of Science	to July 2020	3	Cohort	2 EU;1 Asia	general adults	NR	0-100	19783	4-7	Institution	Iran	1.02(0.94,1.1)	74.3%	low
30238628	Yang MS,2018	fish exposure	highest intake vs lowest intake	2(PubMed and Web of Science	up to April 2018	8	Cohort	3 USA; 2 Australia; 2 Asian; 1 EU	general adults	25-82	0-100	101443(5732 cases	5-25	Fundation	South Korea	0.86(0.76,0.95)	0.0%	low
30238628	Yang MS,2018	fish exposure	1 serving/week increment	2(PubMed and Web of Science	up to April 2018	3	Cohort	1 EU; 1 Australia; 1 Asian	general adults	45-82	0-100	35431(1136 cases	5-25	Fundation	South Korea	0.89(0.75,1.04)	54.8%	low
29031185	Molendijk,2018	fish exposure	highest intake vs lowest intake	3(Embase,PUBMED, and Web of Science	up to March 6th, 2017	6	Cohort	3 EU; 2 USA; 1 Australia	general adults	25-74	38-100	69469	3-31	NR	Netherlands	0.86(0.788,0.96）	68.4%	moderate
26936905	Appleton,2016	n-3 fatty acid intervention	n-3 vs placeBo	3(the Cochrane Depression, Anxiety and Neurosis Review Group’s Specialised Register (CCDANCTR), CINAHL and International Trials Registers	to May 2015	25	RCTs	6 EU; 7 USA; 5 Asia; 2 Canada; 2 Brizal;	depressed adult patients	29-84	0-66	1438		Institution	United Kindom	-0.32(-0.52,-0.12）	58.0%	high
24805797	Grosso, 2014	n-3 fatty acid intervention	n-3 vs placebo	4(MEDLINE, EMBASE, PsycInfo, and the Cochrane Database systematic Reviews"	up to August 2013	19	RCTs	NR	depressed adult patients	NR	NR	1246		institution	Italy	0.56(0.2,0.92)	71.0%	low
26359502	li,2016	fish exposure	highest intake vs lowest intake	3(PubMed, EMBASE and Web of Science	up to March 2015	8	Cohort	2 EU; 3 USA; 1 Asia; 2 Australia;	general adults	18-101	0-100	83087(6175 cases		None	China	0.80(0.72,0.88)	0.0%	low
27544316	Grosso, 2016(1)	fish exposure	highest intake vs lowest intake	4(MEDLINE,EMBASE, PsycInfo, Cochranen Database of Systematic Reviews	up to August 2014	7	Cohort	4 EU; 3 USA	general adults	24-77	NR	111681	4.4-10.6	None	Italy	0.78(0.64,0.93)	53.2%	low
21931319	Bloch,2012	n-3 fatty acid intervention	n-3 vs placebo	1(PUMMED)	up to MAY 2010	13	RCTs	NR	depressed adult patients	NR	NR	731		institution	USA	0.11(-0.04,0.26）	73.0%	low
29031185	Molendijk,2018	Western dietary pattern	highest adherence vs lowest adherence	3(Embase,PUBMED, and Web of Science	up to March 6th, 2017	6	Cohort	2 EU; 2 Australia; 1 USA; 1 Canada	general adults	20-64	0-50	69424	6.5 month-13	NR	Netherlands	1.06 (0.94,1.19)	18.8%	moderate
27544316	Grosso, 2016(1)	fish exposure	50g/d	4(MEDLINE,EMBASE, PsycInfo, Cochranen Database of Systematic Reviews	up to August 2014	2	Cohort	EU	general adults	50-90	NR	12402	4.4-10.6	None	Italy	0.84(0.72,0.99）	Not appliable	low
29759102	Saghafian,2018	fruit exposure	highest intake vs lowest intake	5(Pubmed, ISI (Web of Science), SCOPUS, Embase and Google Scholar"	until Oct 2017	6	Cohort	1 Asia; 2 EU; 2 Australia; 1 USA	general adults	21-85	NR	99224(3726 cases)	NR	institution	Iran	0.83(0.69,0.98)	84.5%	low
29759102	Saghafian,2018	fruit exposure	per 100g increace intake	5(Pubmed, ISI (Web of Science), SCOPUS, Embase and Google Scholar	until Oct 2017	3	Cohort	1 Asian;1 Columbia;1 Australia	general adults	50-79	NR	78855(2593 cases	NR	institution	Iran	0.97(0.95,0.99)	NR	low
26691768	Liu,2016	fruit exposure	highest intake vs lowest intake	3(PubMed, Embase, and Web of Knowledge	up to June 2015	4	Cohort	1 Asia; 1 EU; 1 Australia; 1 North America	general adults	37-79	NR	88949	NR	NR	China	0.8(0.69,0.91)	52.6%	very low

29282023	Zhang,2017	meat consumption	highest intake vs lowest intake	2(PUBMED and EMBASE	up to March 2017	3	Cohort	1 Asia; 1 EU;1 Australia	general adults	37.2-80	0-57.6	20072	NR	foundation	China	1.13(1.03,1.24)	19.4%	very low
30238628	Yang MS,2018	n-3 fatty acid exposure	highest intake vs lowest intake	2(PubMed and Web of Science	up to April 2018	5	Cohort	2 USA; 2 EU; 1 Asian	general adults	24-82	0-100	41952(1544 cases	7.5-25	Fundation	South Korea	0.87(0.74,1.04)	11.0%	low
21939614	Sublette,2011	n-3 fatty acid intervention	n-3 vs placebo	1(PUMMED)	up to June 2010	15	RCTs	NR	depressed adult patients	30-65	NR	916		Fundation	USA	0.55(0.277,0.838)	NR	very low
31004628	Liu,2019	prebiotic intervention	prebiotic vs placebo	3(Embase, MEDLINE, and PsycINFO	NR	5	RCTs	NR	depressed adult patients	23-54	49-51	384		None	USA	-0.08(-0.3,0.15)	NR	very low
31263542	Nikolova, 2019	probiotic strains intervention	probiotic vs placebo	3(PubMed and ISI Google Scholar, Web of Science	on 11 May 2018	3	RCTs	2 Iran; 1Ireland	depressed adult patients	18-55	24%	229		Institution	United Kindom	0.826(-0.527,2.178)	94.7%	very low
30238628	Yang MS,2018	n-3 fatty acid exposure	500mg/d increment	2(PubMed and Web of Science	up to April 2018	4	Cohort	2 USA;1 EU; 1 Asian	general adults	35-82	0-41.6	64134(3377 cases	7.5-25	Fundation	South Korea	0.99(0.94,1.04)	0.0%	low
27544316	Grosso, 2016(1)	n-3 fatty acid exposure	highest intake vs lowest intake	4(MEDLINE,EMBASE, PsycInfo, Cochranen Database of Systematic Reviews	up to August 2014	4	Cohort	3 EU;1 USA	general adults	24-69	NR	41588	2-13	None	Italy	0.84(0.71,0.97)	15.4%	low
27544316	Grosso, 2016(1)	n-3 fatty acid exposure	1.8g/d	4(MEDLINE,EMBASE, PsycInfo, Cochranen Database of Systematic Reviews	up to August 2014	2	Cohort	2 Australia	general adults	25-64	NR	13757	3-10	None	Italy	0.3(0.09,0.98)	Not appliable	low
31004628	Liu,2019	probiotic strains intervention	probiotic vs placebo	3(Embase, MEDLINE, and PsycINFO	NR	23	RCTs	NR	healthy adults	0-100	18-79	2574		None	USA	-0.24(-0.36,-0.12)	48.2%	very low
31647041	Deane,2019	PUFA intervention	higher intake vs lower intake	5(Cochrane Central, Medline and EMBASE,Clinicaltrials.com and the WHO International Clinical Trials Registry Platform	to 27 April,2017	13	RCTs	7 UK; 5 USA; 1 Japan	general adults	50-85	NR	26528(1355 cases)		Fundation	United Kindom	1.01(0.92,1.1)	0.0%	moderate
32937855	Nucci,2020	red and processed meat intake	highest intake vs lowest intake	4(PubMed/Medline, Excerpta Medica dataBASE (EMBASE) and Scopus)	up to March 2020	5	cohort	1 Asia;3 EU;1 Australia	general adults	39-81	NR	21486(2352 cases)	3-9	None	Italy	1.15(1.0,1.3)	48.3%	very low
30419536	Hu,2019	Sugar-sweetened beverages (SSBs) or sweet food consumption	highest intake vs lowest intake	2("PubMed and Web of Science"	up to June 2018	4	Cohort	2 EU; 2 Australia	general adults	35-71	NR	277405(12630 cases)	NR	None	China	1.3(1.19,1.41)	0.0%	low
30419536	Hu,2019	Sugar-sweetened beverages (SSBs) or sweet food consumption	per 2 cup/d of cola	2("PubMed and Web of Science"	up to June 2018	4	Cohort	2 EU; 2 Australia	general adults	35-71	NR	277405(12630 cases)	NR	None	China	1.05(1.01,1.09)	Not appliable	low
29500461	Kang,2018	tea exposure	highest intake vs lowest intake	2(PubMed and Web of Science	to February 2017	5	cohort	3 Asia; 1 EU; 1USA	general adults	42-93	0-100	259818(11937 cases)	NR	Government	South Korea.	0.82(0.48,1.16)	68.7%	very low
26518745	Grosso, 2016(2)	tea exposure	highest intake vs lowest intake	2(PubMed Embase	up to June 2015	2	Cohort	1 Asia; 1 USA	general adults	NR	NR	265538(2073 cases		NR	Italy	1.02(0.73,1.43)	70.0%	low

26518745	Grosso, 2016(2)	tea exposure	100ml/d increment	2(PubMed Embase	up to June 2015	2	Cohort	1Asia; 1 USA	general adults	NR	NR	265538(2073 cases		NR	Italy	0.89(0.64,1.15)	88.7%	low
25657295	Dong,2015	tea exposure	highest intake vs lowest intake	3(Embase, PubMed, and Chinese National Knowledge Infrastructur	through August 2014	5	Cohort	4 Asia;1 EU	general adult population	42-65	NR	9000(1166 cases	NR	None	China	0.81(0.66,0.95)	13.3%	low
25657295	Dong,2015	tea exposure	3 cup/d increment	3(Embase, PubMed, and Chinese National Knowledge Infrastructur	through August 2014	8	3 cohort 5 cross-secttional	7 Asia;1 EU	general adult population	20-76	NR	10600(2107 cases		None	China	0.63(0.55,0.71)	0.0%	low
29759102	Saghafian,2018	vegetable exposure	highest intake vs lowest intake	5(Pubmed, ISI (Web of Science), SCOPUS, Embase and Google Scholar"	until Oct 2017	7	Cohort	2 Asia; 2 EU; 2 Australia; 1 USA	general adults	21-85	NR	100295(4065 cases）	NR	institution	Iran	0.85(0.73,0.97)	68.1%	low
31563280	Goh,2019	probiotic strains intervention	probiotic vs placebo	7(PubMed, Embase, Medline, Cochrane, Web of Science, and ProQuest ; ClinicalTrails.gov/International Clinical Trials Registry Platform	to August 25, 2018.	19	RCTs	10 Asia; 7 EU; 2 New zealand)	general adults	20-85	0-100	1901(intervention 1030; control 871		Institution	China	-0.31(-0.56,-0.07)	86.0%	high
29197739	Qin,2018	probiotic strains intervention	probiotic vs placebo	5(PubMed, Ovid, Clinical Trials Register of the Cochrane Collaboration Depression, Anxiety and Neurosis Group (CCDANTR) and Cochrane Field for Complementary Medicine database	to 1-June-2017	10	RCTs	5 Asia; 3 EU;1 Canada; 1 New zealand	depressed adult patients or healthy adults	18-76	NR	1178		NR	Singapore	-0.128(-0.261,0.00463	0.8%	low
29759102	Saghafian,2018	vegetable exposure	per 100g increace intake	5(Pubmed, ISI (Web of Science), SCOPUS, Embase and Google Scholar"	until Oct 2017	4	Cohort	2 Asia;1 USA ;1 Australia	general adults	50-80	NR	79926(2932 cases	NR	institution	Iran	0.97(0.95,0.98)	NR	low
26691768	Liu,2016	vegetable exposure	highest intake vs lowest intake	3(PubMed, Embase, and Web of Knowledge	up to June 2015	4	Cohort	1 Asia; 1 EU; 1 Australia; 1 North America	general adults	37-79	NR	87795	NR	NR	China	0.87(0.79,0.96)	0.0%	very low
27509521	Huang, 2016	probiotic strains intervention	probiotic vs placeco	7(PubMed, Medline, Springer,Elsevier Science, EMBASE, Cochrane Library, China Knowledge Resource Integrated (CNKI	to 1 January 2016	5	RCTs	3 Asia; 2 EU	healthy and depressed patients	20-65	NR	365(intervention 183; control 182		institution	China	-0.3(-0.51,-0.09）	0.0%	low
30470803	Elin,2019	very low calorie diet	very low calorie vs control	3(PsycINFO, MEDLINE, and Scopus,	to November 10, 2017	11	RCTs	NR	depressed adult patients	20-58	0-53	354		NR	Canada	-0.728(-1.204,-0.253)	NR	very low
24196402	Lai,2014	Western dietary pattern	highest adherence vs lowest adherence	5(Medline, Embase, and PsycInfo via Ovid,EBSCO,Proquest	up to August 2013	2	Cohort	1 EU; 1 USA	general adult population	35-62	0-73.8	54091	5-12	None	Australia	1.25(0.8,1.7)	64.1%	low
28431261	Li Y,2017	western/unhealthy dietary pattern exposure	highest adherence vs lowest adherence	2(MEDLINE and EMBASE databases	up to September 2016	8	Cohort	2 Asia; 3 EU; 1 Australia; 1 Canada; 1 USA	general adults	20-77	NR	75481	NR	Government	China	1.16(1.01,1.34)	71.0%	low

25701329	Gowda M.H.N. ,2015	vitamin D intervention	vitamin D vs placebo	5(MEDLINE, EMBASE, psych INFO, CINAHL plus, and Cochrane library	to May 2014	9	RCTs	2 EU; 3 USA; 3 Australia; 1 Asia	general adult population	22-72	NR	4923		Fundation	Australia	0.28(-0.14,0.69）	85.0%	low
24632894	Shaffer, 2014	vitamin D intervention	vitamin D vs placebo	6(Ovid MEDLINE, The Cochrane Library, Cumulative Index to Nursing and Allied Health Literature, Allied and Complimentary Medicine Database, PsycINFO, and Scopus	to the second week of May,2013	7	RCTs	2 USA; 2 EU; 2 Asia; 1 Australia	depressed adult patients	18-70	0-46	3191(intervention 1640 control 1551)		Fundation	USA	-0.14(-0.33,0.05)	70.3%	low
24423304	Li,2014	vitamin D intervention	vitamin D vs placebo	6(Ovid MEDLINE, The Cochrane Library, Cumulative Index to Nursing and Allied Health Literature, Allied and Complimentary Medicine Database, PsycINFO, and Scopus	up to April 2013	6	RCTs	2 USA; 2 EU; 1 Asia; 1 Australia	depressed adult patients	18-70	0-46	1203		None	China	-0.14(-0.41,0.13)	77.0%	high
