# Supplementary material for: Role of dietary factors in the prevention and treatment for depression: an umbrella review of meta-analyses of prospective studies
Source: Transl Psychiatry. 2021 Sep 16;11:478. doi: 10.1038/s41398-021-01590-6 (PMC8445939; doi:10.1038/s41398-021-01590-6)
Supplement: Supplementary file 3 — Supplementary table 3 [file 41398_2021_1590_MOESM3_ESM.pdf]

Supplementary Table 3. The detail scores of AMSTAR-2 for meta-analyses included in the umbrella review.

Author (year)	Critical domains							non-critical domains									
	Established methods prior to conduction	Comprehensive literature search strategy	List of excluded studies and justify them	Suitable technique for risk bias assessment	Appropriate methods for statistical combination of results	Disscussion on risk of bias	Investigation of publication bias and discussion	PICO components	Study design selection explanation	Study selection in duplicate	Data extraction in duplicate	Describe included studies in detail	Report on the funding of primary studies	Assessment on the risk of bias for synthesis	explanation or discussion for heterogeneity	Report on conflict of interest	Level
Pagliai,2021	YES	YES	YES	YES	YES	YES	YES	YES	YES	YES	YES	YES	NO	YES	YES	YES	high
Tome,2021	PARTLY YES	YES	YES	YES	YES	YES	YES	YES	YES	YES	YES	YES	NO	YES	YES	YES	low
Fusar-Poli,2021	YES	YES	YES	YES	YES	YES	YES	YES	YES	YES	YES	YES	NO	YES	YES	YES	high
Yosaee, 2020	YES	YES	YES	YES	YES	YES	YES	YES	YES	YES	YES	YES	NO	YES	YES	YES	high
Nucci,2020	NO	YES	YES	YES	YES	YES	YES	YES	YES	YES	YES	YES	NO	YES	YES	YES	low
Li,2020	PARTLY YES	YES	YES	YES	YES	YES	YES	YES	YES	YES	NO	YES	NO	YES	YES	YES	low
Askari,2020	NO	YES	YES	YES	YES	YES	YES	YES	YES	NO	YES	YES	NO	YES	YES	YES	low
Young,2019	NO	YES	YES	NO	YES	NO	NO	YES	YES	YES	YES	YES	NO	NO	YES	YES	very low
Tolkien,2019	PARTLY YES	PARTLY YES	YES	YES	YES	YES	YES	YES	YES	YES	NO	YES	NO	YES	YES	YES	very low
Shafiei,2019	NO	YES	YES	YES	YES	YES	YES	YES	YES	YES	YES	YES	NO	YES	YES	YES	low
Liu,2019	NO	YES	YES	YES	YES	YES	YES	YES	YES	NO	NO	YES	NO	YES	YES	YES	low
Liao,2019	NO	YES	YES	YES	YES	YES	YES	YES	YES	YES	YES	YES	NO	YES	YES	YES	low
Lassale,2019	YES	YES	YES	YES	YES	YES	YES	YES	YES	NO	NO	YES	NO	YES	YES	YES	Moderate
Hu,2019	NO	YES	YES	YES	YES	YES	YES	YES	YES	YES	YES	YES	NO	YES	YES	YES	low
Firth,2019	YES	YES	YES	YES	YES	YES	YES	YES	YES	YES	YES	YES	NO	YES	YES	YES	high
Elin,2019	NO	PARTLY YES	YES	NO	YES	NO	YES	YES	YES	YES	YES	YES	NO	NO	YES	YES	very low
Deane,2019	YES	YES	YES	YES	YES	YES	YES	YES	YES	YES	YES	YES	NO	YES	YES	YES	high
Yang MS,2018	NO	YES	YES	YES	YES	YES	YES	YES	YES	NO	YES	YES	NO	YES	YES	YES	low
Veronese,2018	NO	YES	YES	YES	YES	YES	YES	YES	YES	NO	YES	YES	NO	YES	YES	YES	low
Saghafian,2018	NO	YES	YES	YES	YES	YES	YES	YES	YES	YES	NO	YES	NO	YES	YES	YES	low
Molendijk,2018	YES	YES	YES	YES	YES	YES	YES	YES	YES	NO	NO	YES	NO	YES	YES	YES	moderate
Kang,2018	NO	YES	YES	NO	YES	NO	YES	YES	YES	NO	YES	YES	NO	YES	YES	YES	very low
Li Y,2017	NO	YES	YES	YES	YES	YES	YES	YES	YES	YES	NO	YES	NO	NO	YES	YES	low
Li BR,2017	NO	YES	YES	NO	YES	NO	YES	YES	YES	YES	NO	YES	NO	YES	YES	YES	very low
Wang,2016	NO	YES	YES	NO	YES	NO	YES	YES	YES	YES	NO	NO	NO	YES	YES	YES	very low
Sarris,2016	NO	YES	YES	NO	YES	NO	YES	YES	YES	NO	NO	YES	NO	YES	YES	YES	very low
Grosso,2016(1)	NO	YES	YES	YES	YES	YES	YES	YES	YES	YES	YES	YES	NO	YES	YES	YES	low
Grosso,2016(2)	NO	YES	YES	YES	YES	YES	YES	YES	YES	YES	YES	YES	NO	YES	YES	YES	low
