# Supplementary material for: Role of dietary factors in the prevention and treatment for depression: an umbrella review of meta-analyses of prospective studies
Source: Transl Psychiatry. 2021 Sep 16;11:478. doi: 10.1038/s41398-021-01590-6 (PMC8445939; doi:10.1038/s41398-021-01590-6)
Supplement: Supplementary file 4 — Supplementary table 4 [file 41398_2021_1590_MOESM4_ESM.pdf]

Supplementary Table 4. The detail scores in the NutriGrade for included meta-analyses in this umbrella review.

PMID	Author (year)	Dietary intervention/exposures	Comparion	Risk of bias	Precision	Heterogeneity	Directness	Publication bias	Funding bias	Effect size	Dose-response	Overall scores	level
32792031	Pagliai,2021	ultra-processed foods	highest intake vs lowest intake	2	1	0	1	0	1	0	0	5	low
33970709	Fusar-Poli, 2021	cocoa-rich foods	cocoa-rich products vs placebo	2	1	0	1	0	1	2		7	moderate
33533015	Tome, 2021	vitamin D supplementation	vitamin D vs placebo	2	0	0.8	1	1	1	2		7.8	moderate
32829928	Yosaee, 2020	dietary zinc exposure	highest intake vs lowest intake	2	1	0	1	0	1	1	0	6	Moderate
32829928	Yosaee, 2020	dietary zinc supplementation	zinc vs placebo/antidepressants	2	0	0.4	0	0.5	1	2		5.9	low
32937855	Nucci,2020	red and processed meat intake	highest intake vs lowest intake	2	1	0	1	0.5	0.5	0	0	5	low
31837230	Li,2020	drink intake	heavy drinking vs non-heavy	1	1	0.8	1	1	1	0	1	6.8	moderate
32885996	Askari,2020	vegetarian diet	highest adherence vs lowest adherence	2	0	0	1	0	1	0	0	4	low
31527485	Young,2019	B vitamin supplementation	B vitamin vs placebo	0	0	0.4	0	0.5	1	2	0	3.9	very low
30502975	Tolkien,2019	inflammatory potential of the diet（DII or cytokine）	higherstadherence vs lowest adherence	1	1	0.4	1	0.5	0.5	1	0	5.4	low
30726966	Shafiei,2019	Mediterranean diet	highest adherence vs lowest adherence	2	0	0	1	0	1	0	0	4	low
31004628	Liu,2019	prebiotic intervention	prebiotic vs placebo	1	0	0	0	0.5	1	2		4.5	low
31383846	Liao,2019	n-3 fatty acid intervention	n-3 fatty acid vs placebo	1	1	0.8	0	1	1	2		6.8	moderate
30254236	Lassale,2019	AHEI or AHEI-2010 scores	highest adherence vs lowest adherence	2	1	0	1	0	1	1	0	6	moderate
30419536	Hu,2019	Sugar-sweetened beverages (SSBs) or sweet food consumption	highest intake vs lowest intake	2	1	0	1	0	0.5	1	1	6.5	moderate
31004628	Liu,2019	probiotic strains intervention	probiotic vs placebo	1	1	0.8	0	1	1	2		6.8	moderate
30720698	Firth,2019	dietary intervention	dietary intervention vs non dietary control	2	1	0.8	0	1	1	2		7.8	moderate
30470803	Elin,2019	very low calorie diet	very low calorie vs control	0	1	0.4	0	1	0.5	2		4.9	low

31647041	Deane,2019	PUFA intervention	higher intake vs lower intake	1	1	0.8	0	1	1	2		6.8	moderate
30238628	Yang MS,2018	fish exposure	higher intake vs lower intake	2	1	0.4	1	0.5	1	0	1	6.9	moderate
29076953	Veronese,2018	Acetyl-L-Carnitine supplementation intervention	ALC vs placebo	1	1	0.3	1	0.5	0.5	2		6.3	moderate
29076953	Veronese,2018	Acetyl-L-Carnitine supplementation intervention	ALC vs antidepressant	2	0	0	1	0	0.5	2		5.5	low
29759102	Saghafian,2018	fruit exposure	highest intake vs lowest intake	1	1	0.4	1	0.5	1	0	1	5.9	low
29759102	Saghafian,2018	vegetable exposure	highest intake vs lowest intake	1	1	0.5	1	0.5	1	0	0	5	low
29031185	Molendijk,2018	healthy dietary pattern	highest adherence vs lowest adherence	2	1	0.4	1	0.5	0.5	1	0	6.4	moderate
29500461	Kang,2018	tea exposure	highest intake vs lowest intake	0	1	0.3	1	0.5	1	0	0	3.8	very low
28431261	Li Y,2017	western/unhealthy dietary pattern exposure	highest adherence vs lowest adherence	2	0	0.4	1	0.5	1	0	0	4.9	low
27807012	Li BR,2017	dietary magnium exposure	highest intake vs lowest intake	0	1	0	1	0	0.5	0	1	3.5	very low
26339067	Wang,2016	caffeine exposure	highest intake vs lowest intake	0	1	0	1	0	1	0	1	4	low
27113121	Sarris,2016	folic acid supplementation	folic acid vs placebo	0	0	0	1	0	0.5	2		3.5	very low
27544316	Grosso,2016(1)	n-3 fatty acid exposure	highest intake vs lowest intake	2	1	0	1	0	0.5	0	1	5.5	low
26518745	Grosso,2016(2)	coffee exposure	highest intake vs lowest intake	2	1	0	1	0	0.5	1	1	6.5	moderate
