# Supplementary material for: Role of dietary factors in the prevention and treatment for depression: an umbrella review of meta-analyses of prospective studies
Source: Transl Psychiatry. 2021 Sep 16;11:478. doi: 10.1038/s41398-021-01590-6 (PMC8445939; doi:10.1038/s41398-021-01590-6)

Supplementary Table 5. Funnel plot for meta-analyses with more than 5 primary studies.

A. Funnel plot for the dietary pattern. A1, for DII exposure; A2, for healthy dietary pattern exposure; A3, for western dietary pattern exposure; A4, for healthy diet intervention; A5, for low calorie diet intervention.

A1. Tolkien, 2019, PMID: 30502975

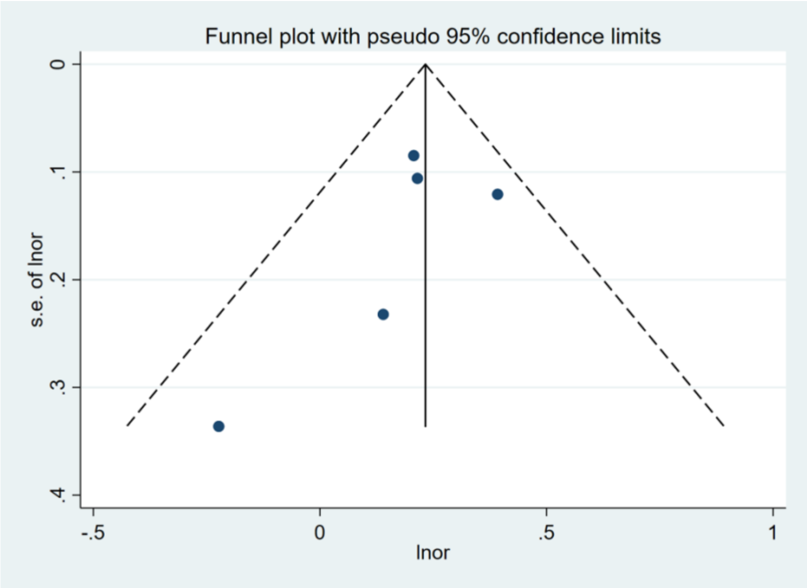

A2. Molendijk, 2018, PMID: 29031185

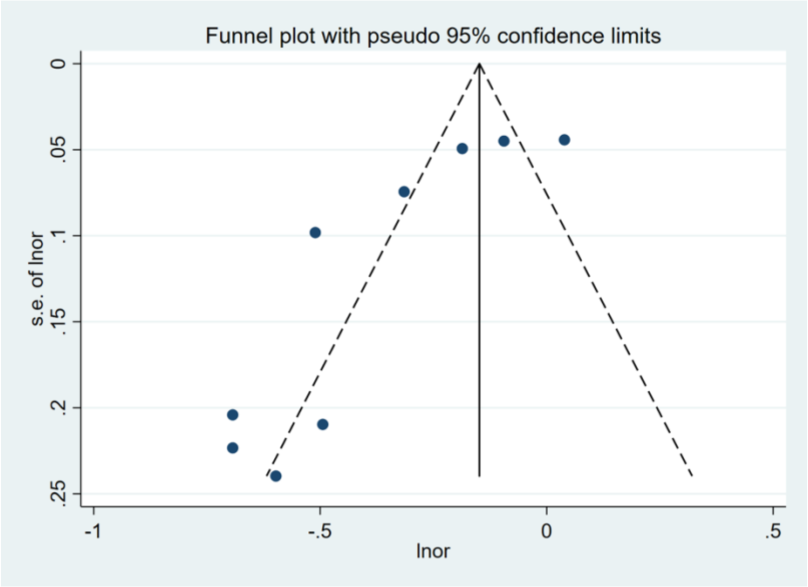

A3. Li Y, 2017, PMID: 28431261

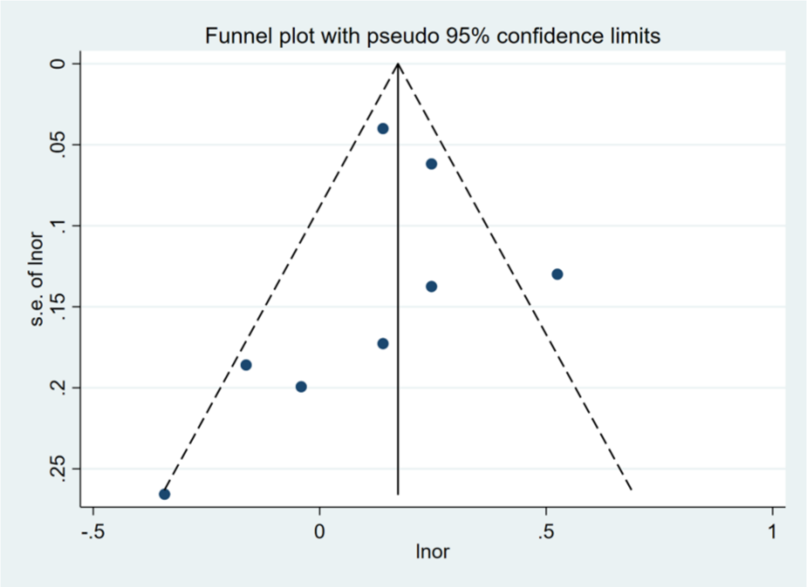

A4. Firth, 2019, PMID: 31004628

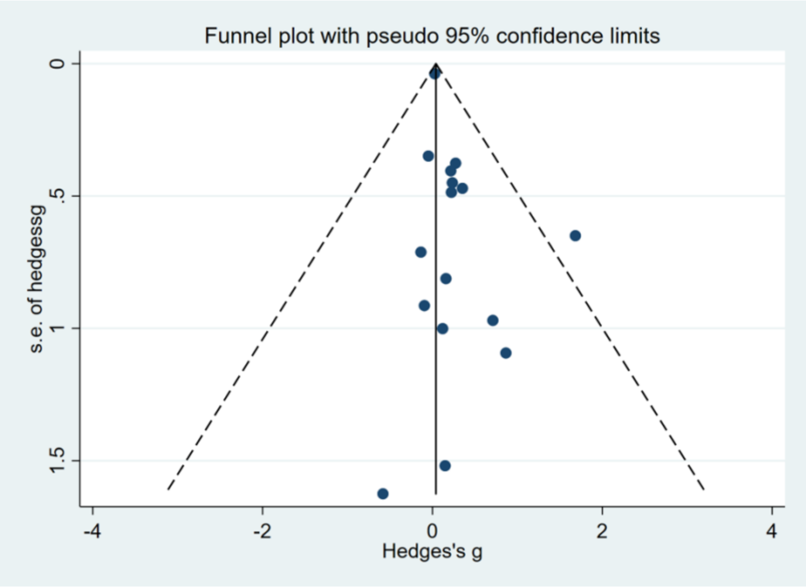

A5. Elin, 2019, PMID: 30470803

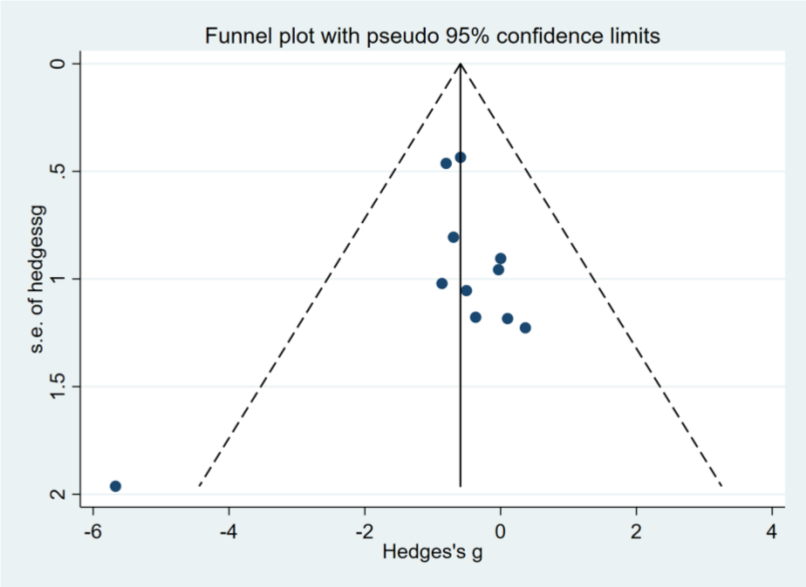

B. Funnel plot for the food groups. B1, for red and processed meat intake , B2, for alcohol drink, B3, for fish intake, B4, for fruit intake, B5, for vegetable intake, B6, for tea intake, B7, for cocoa-rich foods intervention, B8, for probiotics intervention.

B1. Nucci, 2020, PMID: 32937855

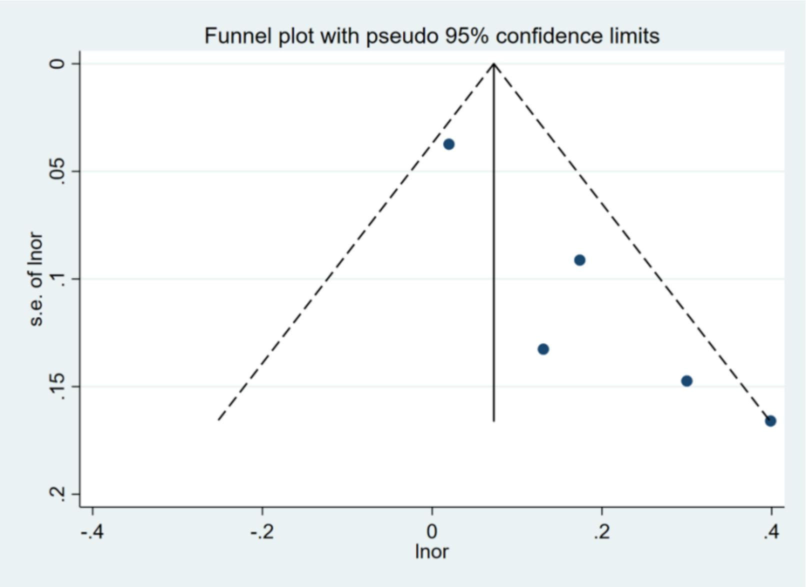

B2. Li, 2020, PMID: 31837230

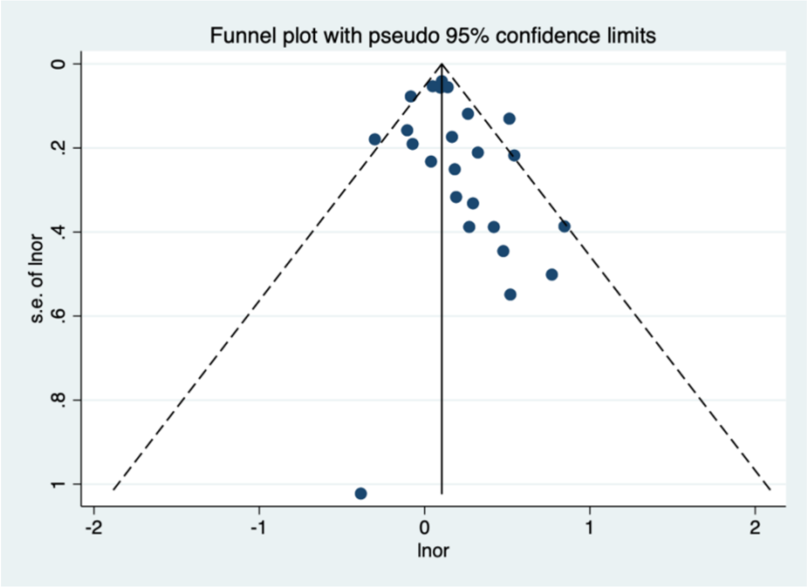

B3. Yang MS, 2018, PMID: 30238628

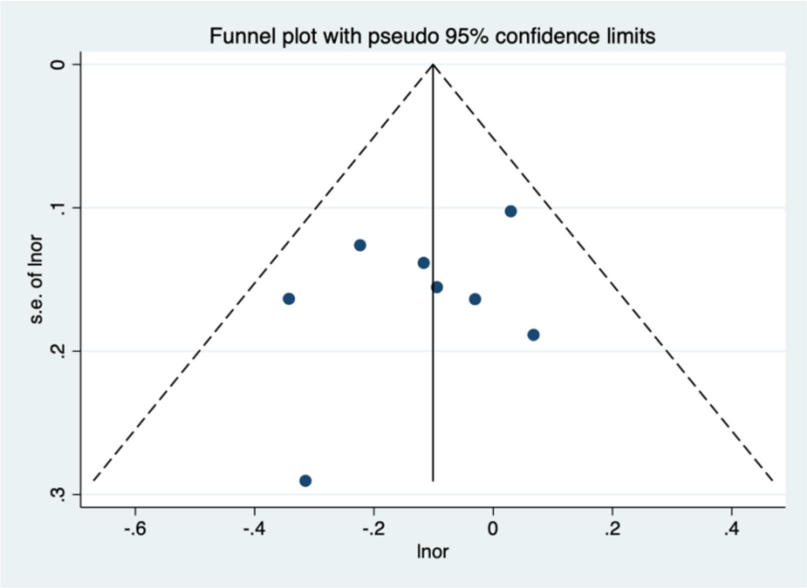

B4. Saghafian, 2018, PMID: 29759102

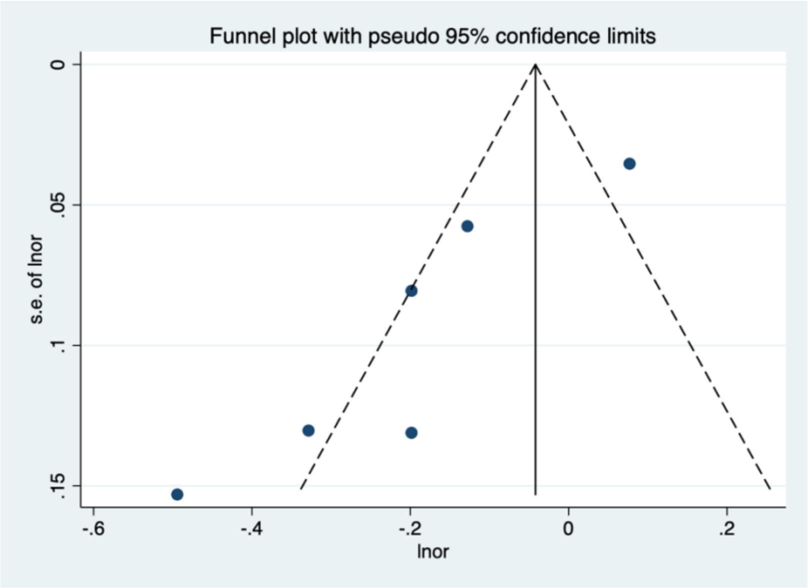

B5. Saghafian, 2018, PMID: 29759102

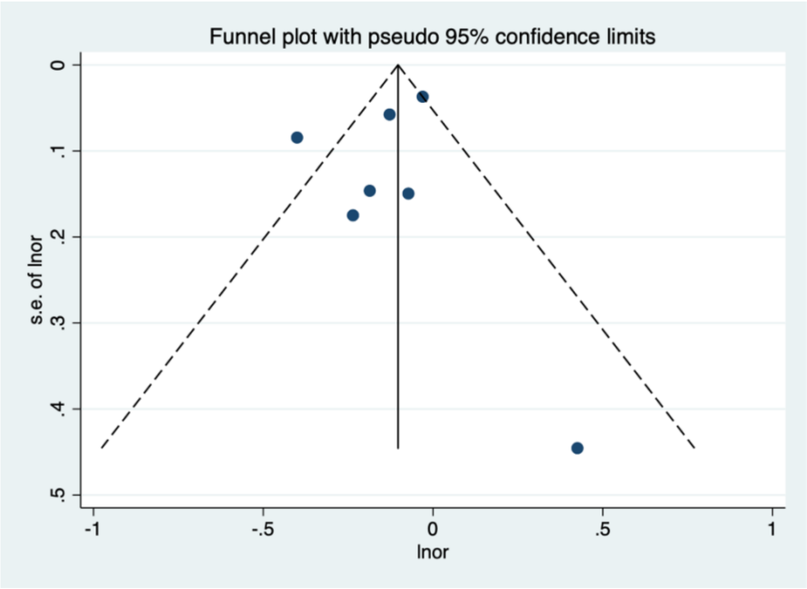

B6. Kang, 2018, PMID: 29500461

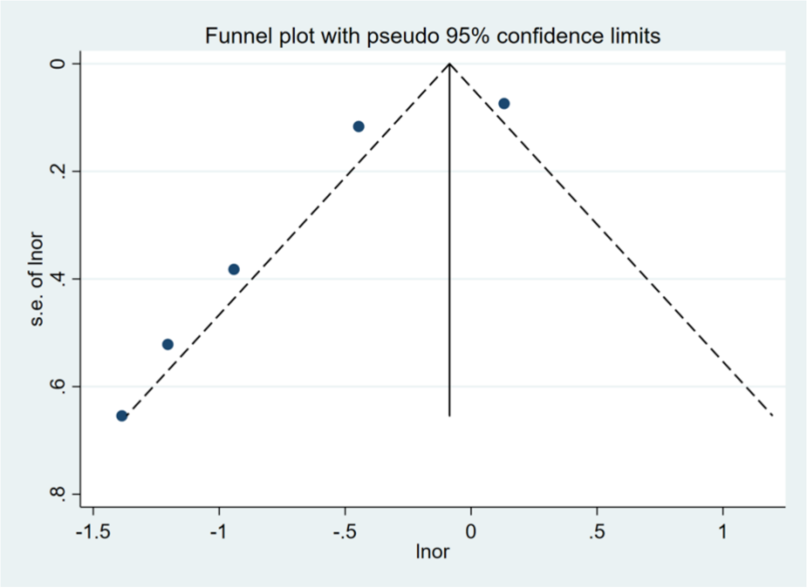

B7. Fusar-Poli, 2021, PMID: [33970709]

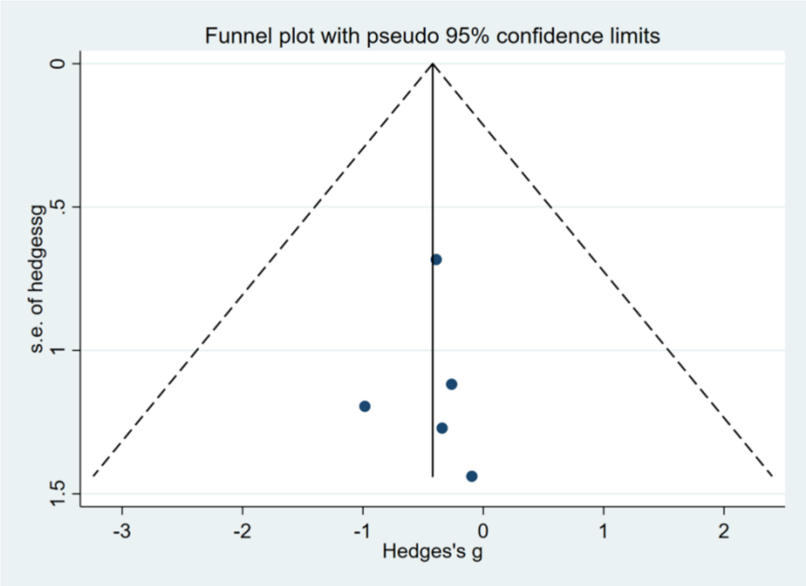

B8. Liu, 2019, PMID: [31004628]

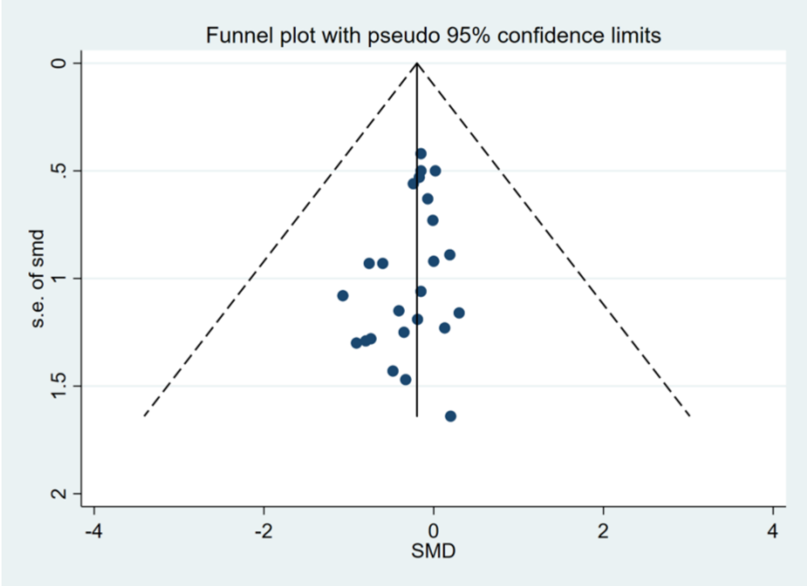

C. Funnel plot for the nutrients. C1, for n-3 PUFA intake , C2, for vitamin D intervention, C3, for dietary zinc intervention, C4, for B vitamins intervention, C5, for n-3 PUFA intervention, C6, for ALC intervention.

C1. Deane,2019, PMID: 31647041

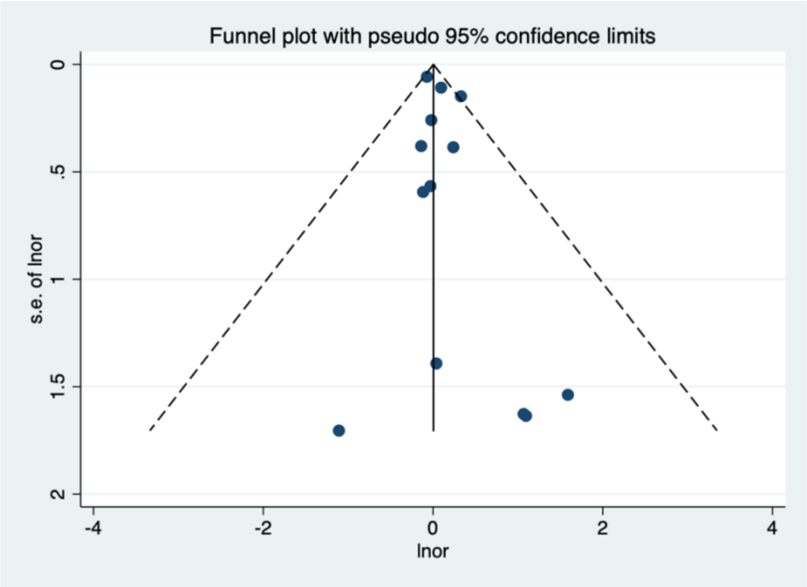

C2. Tome, 2021, PMID: 33533015

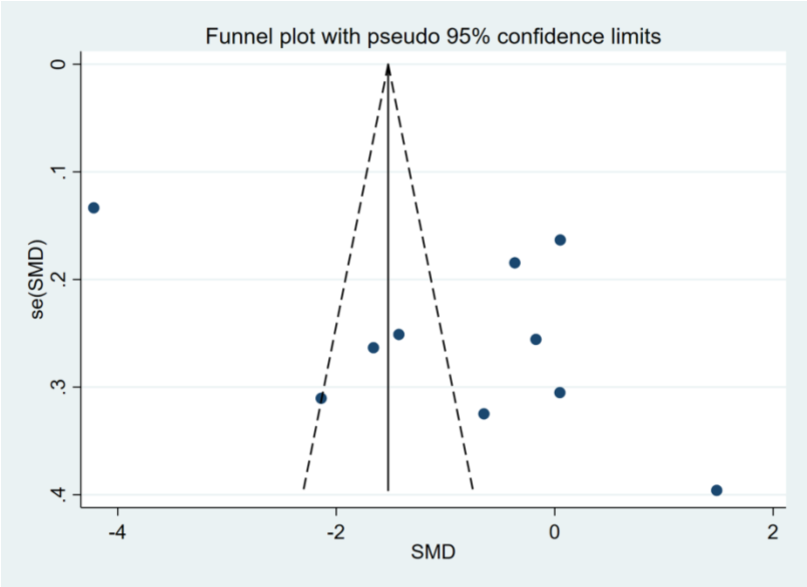

C3. Yosaee, 2020, PMID: 32829928

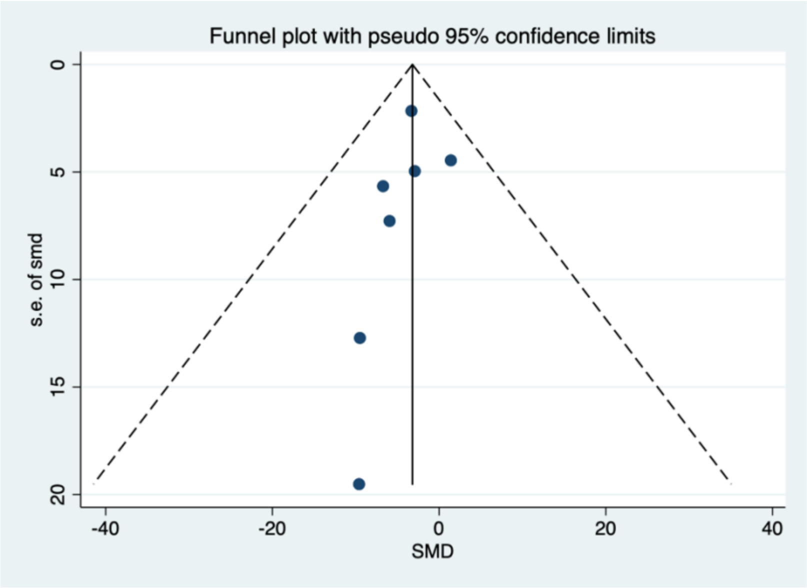

C4. Young, 2019, PMID: 31527485

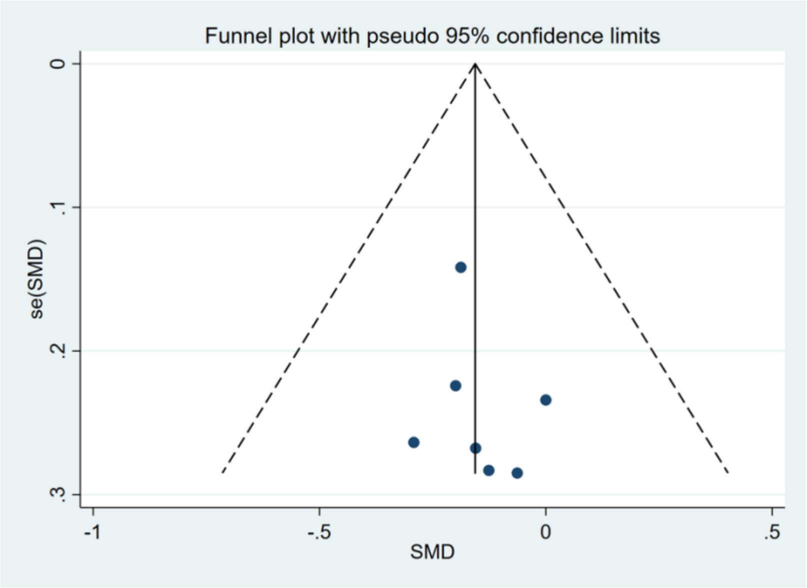

C5. Liao,2019, PMID: 31383846

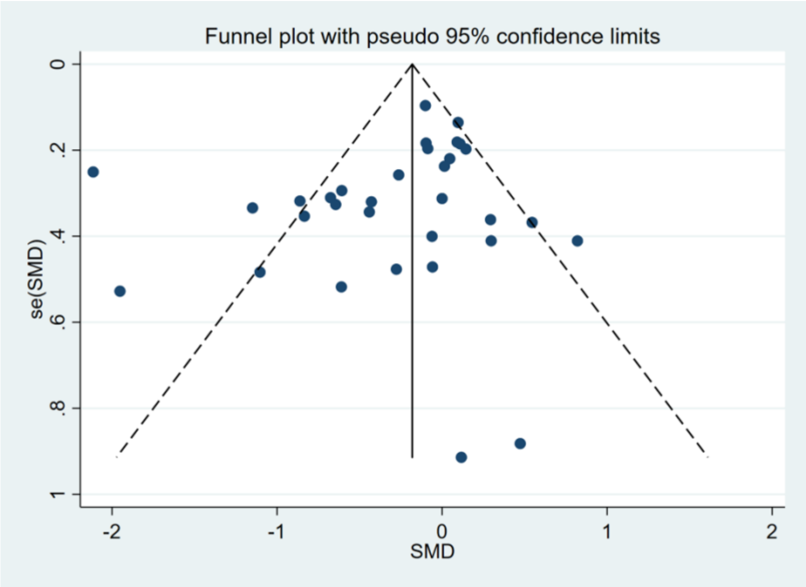

C6. Veronese, 2018, PMID: 29076953

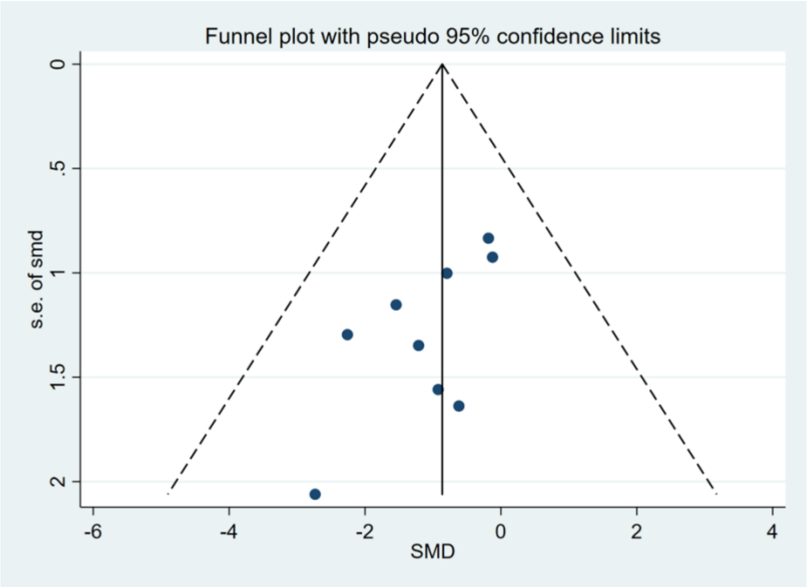

Supplement: Supplementary file 5 — Supplementary table 5 [file 41398_2021_1590_MOESM5_ESM.pdf]
